# Physiological and transcriptomic analysis dissects the molecular mechanism governing meat quality during postmortem aging in Hu sheep (*Ovis aries*)

**DOI:** 10.3389/fnut.2023.1321938

**Published:** 2024-01-05

**Authors:** Huan Li, Yan-Hui Feng, Chao Xia, Yu Chen, Xin-Yi Lu, Yue Wei, Le-Le Qian, Meng-Yao Zhu, Guo-Yv Gao, Ya-Fei Meng, Yv-Le You, Qi Tian, Kun-Qi Liang, Yun-Tao Li, Chao-Tian Lv, Xiang-Yun Rui, Ming-Yue Wei, Bin Zhang

**Affiliations:** ^1^College of Food and Bio-engineering, Bengbu University, Bengbu, Anhui, China; ^2^College of Food Engineering, Anhui Science and Technology University, Chuzhou, Anhui, China; ^3^School of Ecology, Resources and Environment, Dezhou University, Dezhou, Shandong, China

**Keywords:** lamb meat, post-slaughter storage, aging process, transcript profiles, regulatory network

## Abstract

**Introduction:**

Hu sheep, known for its high quality and productivity, lack fundamental scientific research in China.

**Methods:**

This study focused on the effects of 24 h postmortem aging on the meat physiological and transcriptomic alteration in Hu sheep.

**Results:**

The results showed that the 24 h aging process exerts a substantial influence on the mutton color, texture, and water content as compared to untreated group. Transcriptomic analysis identified 1,668 differentially expressed genes. Functional enrichment analysis highlighted the importance of glycolysis metabolism, protein processing in endoplasmic reticulum, and the FcγR-mediated phagocytosis pathway in mediating meat quality modification following postmortem aging. Furthermore, protein-protein interaction analysis uncovered complex regulatory networks involving glycolysis, the MAPK signaling pathway, protein metabolism, and the immune response.

**Discussion:**

Collectively, these findings offer valuable insights into the molecular mechanisms underlying meat quality changes during postmortem aging in Hu sheep, emphasizing the potential for improving quality control strategies in mutton production.

## 1 Introduction

Mutton holds great value in Chinese culinary culture and offers numerous benefits. Its exceptional taste, high protein content, balanced fat levels, and low cholesterol levels contribute to its consumer appeal (1). Consequently, there is a growing demand for lamb meat. Additionally, the improvement in living standards and changes in dietary preferences have led consumers to expect better quality mutton. However, meeting these expectations poses challenges due to the large market supply and demand. Therefore, it becomes essential to conduct thorough research on the post-slaughter quality of mutton.

Postmortem aging is an essential stage in mutton processing, and extensive research has demonstrated its significant enhancement of meat quality (2–5). This improvement is primarily due to anaerobic respiration during post-slaughter storage, resulting in the generation of lactic acid. Under controlled conditions of temperature, humidity, and airflow, lactic acid is enzymatically decomposed into carbon dioxide and water. And the intracellular adenosine triphosphate is hydrolyzed into the flavor-enhancing substance inosine monophosphate. Moreover, the altered pH not only increases tenderness but also aids digestion and absorption. Edible quality is a key determinant of meat quality, focusing on sensory attributes such as color, tenderness, and water retention. The duration of maturation after slaughter significantly affects the edible quality of the meat. Abdullah and Qudsieh (2) studied the quality changes in mutton stored for 24 and 168 h, and found that the meat within 168 h-treated exhibited increased brightness (L*) and redness (a*), as well as improved tenderness. Gao et al. (4) found that Jinta mutton that underwent aging process under optimal conditions (0–4°C, 90% humidity, hung for 16–20 h, then frozen at −20°C and matured on the second day) had superior meat color, pH, and flavor compared to untreated mutton. Martínez-Cerezo et al. (5) observed that postmortem aging affected the texture of Rasa Aragonesa, Churra, and Spanish Merino lambs, especially in the first 4 days, and the effect continued until day 16, but with a slower tenderization rate. In addition, Choe et al. (3) found that increasing the aged temperature (3 or 7°C) for lamb loin significantly shortened the required treatment time before freezing while maintaining equivalent quality characteristics, such as tenderness, drip loss, and shelf life, as 14 days aged-treated loin at −1.5°C. Despite significant research on the physiological and biochemical effects of postmortem aging on mutton, further investigation is needed to decipher the underlying transcriptional expression profiles and molecular mechanisms.

Transcriptomics is an effective means of studying gene expression and regulatory patterns to reveal biological pathways and molecular mechanisms. It is widely used in animal research, especially for identifying candidate genes related to meat quality in livestock. For instance, Fernández-Barroso et al. (6) identified 200 differentially expressed genes and 245 novel isoforms in Iberian pigs with varying tenderness levels. Muniz et al. (7) using RNA-Sequencing (RNA-Seq) found newly mRNA isoforms linked to beef tenderness, involving oxidative processes, energy production, and striated muscle contraction. Damon et al. (8) discovered that breed differences in pigs’ muscle gene expressions and chemical composition are linked to energy metabolism, lipid deposition, and the role of cytoskeleton and contractile fibers in determining muscle and meat phenotypes. This technology has also been applied to research in mutton. Miao et al. (9) observed significant down-regulation of metabolic processes, particularly lipid metabolism, in Small Tail Han sheep’s adipose tissue compared to Dorset sheep, potentially explaining disparities in fat deposition. Moreover, mitochondrial genes ATP synthase F0 subunit 6, cytochrome c oxidase subunit I, II, and cytochrome b were identified as core tenderness-related genes in Tan sheep meat (10). RNA-Seq, therefore, offers robust technical support for understanding molecular mechanisms after postmortem aging of mutton at the transcriptional level.

The Hu sheep (*Ovis aries*) of China is well-known for its early maturity, high productivity, and excellent meat production (11). This local variety, with a breeding history of over 800 years, also shows resilience to high temperatures and humidity (12). It is preferred for factory-scale meat sheep production and its market sales are on the rise. However, research on Hu sheep is still in its early stages, indicating significant potential for development. Thus, this study was performed to explore the potential mechanisms related to meat quality during postmortem aging using physiological, transcriptomic, and bioinformatic approaches.

## 2 Materials and methods

### 2.1 Sample preparation and treatment

In October 2022, nine 6-month-old male Hu sheep (*O. aries*) from a pasture located at Anhui Zhenghua Yang Ye Co., Ltd. in China, with comparable body weights (45 ± 1.62 kg) and feeding protocols, were selected for this study. Fresh lamb muscles between the 12th and 13th ribs on either side were meticulously packed in an insulated container and transported to the laboratory under controlled temperature conditions ranging between 0 and 4°C (13). The Hu sheep meat batch was then segregated into two distinct groups: a control group designated as before the aging process (BA) and an experimental group referred to as after the aging process (AA). The Hu sheep meat samples in the BA group underwent no treatment and were directly collected for index testing. On the other hand, the Hu sheep meat samples in the AA group underwent a refrigerated aging process at a temperature of 4°C for 24 h prior to conducting the assays. A portion of the Hu sheep meat samples were selected for physiological analysis such as color and pH measurements, determination of textural profile and cooking loss, and assessment of nuclear magnetic properties. The remaining samples weighing 0.2 g were immediately cryopreserved in liquid nitrogen for future transcriptome sequencing studies, with three biological repeats.

### 2.2 Color and pH measurement

To ensure accurate color measurement, only areas without any apparent imperfections that could potentially impact the consistency of color readings were selected (14). To measure meat color, the surface of meat samples was scanned using an NR10QC Color Meter from Shenzhen ThreeNH Technology Co., Ltd., which provided readings for lightness (L*), redness (a*), and yellowness (b*) in accordance with the manufacturer’s instructions. pH values in the muscle tissue were measured by inserting a calibrated pH probe (PHs-2F from Shanghai INESA Scientific Instrument Co., Ltd.) to a depth of 2 cm in postmortem meat at random locations (15).

### 2.3 Textural profile determination and cooking loss analysis

The TA. XT Express texture analyzer (Stable Micro Systems, United Kingdom) was used to analyze the texture of the Hu sheep meat before and after the aging process, following the method of Dong et al. (11) with slight modifications. Hardness (N) and chewiness (mJ) values were obtained by setting the parameters of the texture analyzer, including a pre-test speed of 5 mm/s, a test speed of 1.5 mm/s, a post-test speed of 5 mm/s, a 5-s pause time between compressions, and a trigger force of 5 g. The cooking loss is performed with the method of Wang et al. (12). In brief, high-quality meat samples were precisely weighed and measured to ensure a consistent thickness. A thermometer was inserted into the center of each sample and sealed in a steaming bag. The samples were heated in a controlled 80°C water bath until the core temperature reached 75°C. The heating process was stopped after 20 min of constant temperature. Once cooled to room temperature, the surface was carefully dried, and each sample was weighed. The cooking loss was determined by quantifying the weight difference of Hu sheep meat before and after cooking, under both BA and AA treatment conditions. The resulting values were then expressed as a percentage (%).

### 2.4 Magnetic resonance imaging measurement

Magnetic resonance imaging (MRI) measurements were conducted based on the method of Dong et al. (11) with alterations. The sample was placed in the center of the coil for MRI testing. A SPIN ECHO sequence was employed and the main parameters of the magnetic imaging (MRI) were average = 2, slice width (mm) = 3.0, slice gap (mm) = 2.0, waiting time (TR) = 500 ms, echo time (TE) = 20 ms, phase size = 192, and read size = 256.

### 2.5 Low-field nuclear MR analysis

The acquisition of transverse (T_2_) relaxation in Low-field nuclear MR (LF-NMR) was carried out following the method of Zheng et al. (16), with modifications. Water distribution of the Hu sheep meat samples was measured with the NMR analysis software. A sample of approximately 2 g of Hu sheep meat was placed in the center of the coil for nuclear magnetic testing, and the center frequency of the sample was obtained by an FID sequence. The CPMG sequence was then used for the subsequent detection. The main parameters of the T_2_ test included SF (MHz) = 21, RFD (ms) = 0.02, O_1_ (Hz) = 328,606.75, RG1 (db) = 10.0, P1 (μs) = 14.00, DRG1 = 3, TD = 120,012, PRG = 3, TW (ms) = 4,500.00, NS = 16, TE (ms) = 0.200, and NECH = 3,000. After information was collected, data inversion was performed to obtain the transverse relaxation time T_2_ of the sample.

### 2.6 RNA extraction, sequencing, and data processing

The samples of Hu sheep meat underwent cryogenic grinding in liquid nitrogen. Total RNA was extracted from each sample using a Quick RNA isolation kit (Bioteke Corporation, Beijing, China). The quality assessment of the extracted total RNA was carried out using the NanoDrop 2000 (Thermo Fisher Scientific, USA), Qubit 2.0 fluorometer (Life Technologies, USA), and Agilent 2,100 bioanalyzer (Agilent, USA) (17). Subsequently, the total RNA was sent to Beijing Novel Bioinformatics Co., Ltd. for the construction of cDNA libraries and *de novo* transcriptome sequencing using the Illumina NovaSeq 6,000 platform, which resulted in the generation of 150 bp paired-end reads (18). The raw sequencing data were archived in the Sequence Read Archive with the accession number SRR25065590-SRR25065595. After rigorous quality control, the clean reads were utilized for subsequent analyses. Differential gene expression analysis was performed using FPKM values and DESeq2 (19, 20). Genes meeting the criteria of having a value of *p* < 0.05 and |log2FoldChange| > 0 were deemed as differentially expressed genes (DEGs) (21, 22). The potential molecular functions and biochemical pathways were determined via Gene Ontology (GO) enrichment analysis with a significance threshold of value of *p* < 0.05, and Kyoto Encyclopedia of Genes and Genomes (KEGG) metabolic pathway enrichment analysis with a corrected value of *p* < 0.01, following the method of Young et al. (23) and Mao et al. (24).

### 2.7 Statistical analysis

The One-way ANOVA analysis, followed by Duncan’s multiple range test with a significance threshold of *p* < 0.05, was used to perform significance analysis on the data presented in the figures and tables of the experiment using SPSS software (Version 21) (18). The means ± SE were used to represent all data depicted in the figures. Venn diagrams and hierarchical clustering were analyzed on the Novogene online platform.^1^ Additionally, other graphical representations were generated using GraphPad Prism software (Version 9.0.2).

## 3 Results

### 3.1 Changes in mutton color and pH of Hu sheep after postmortem aging

**Figure 1:**
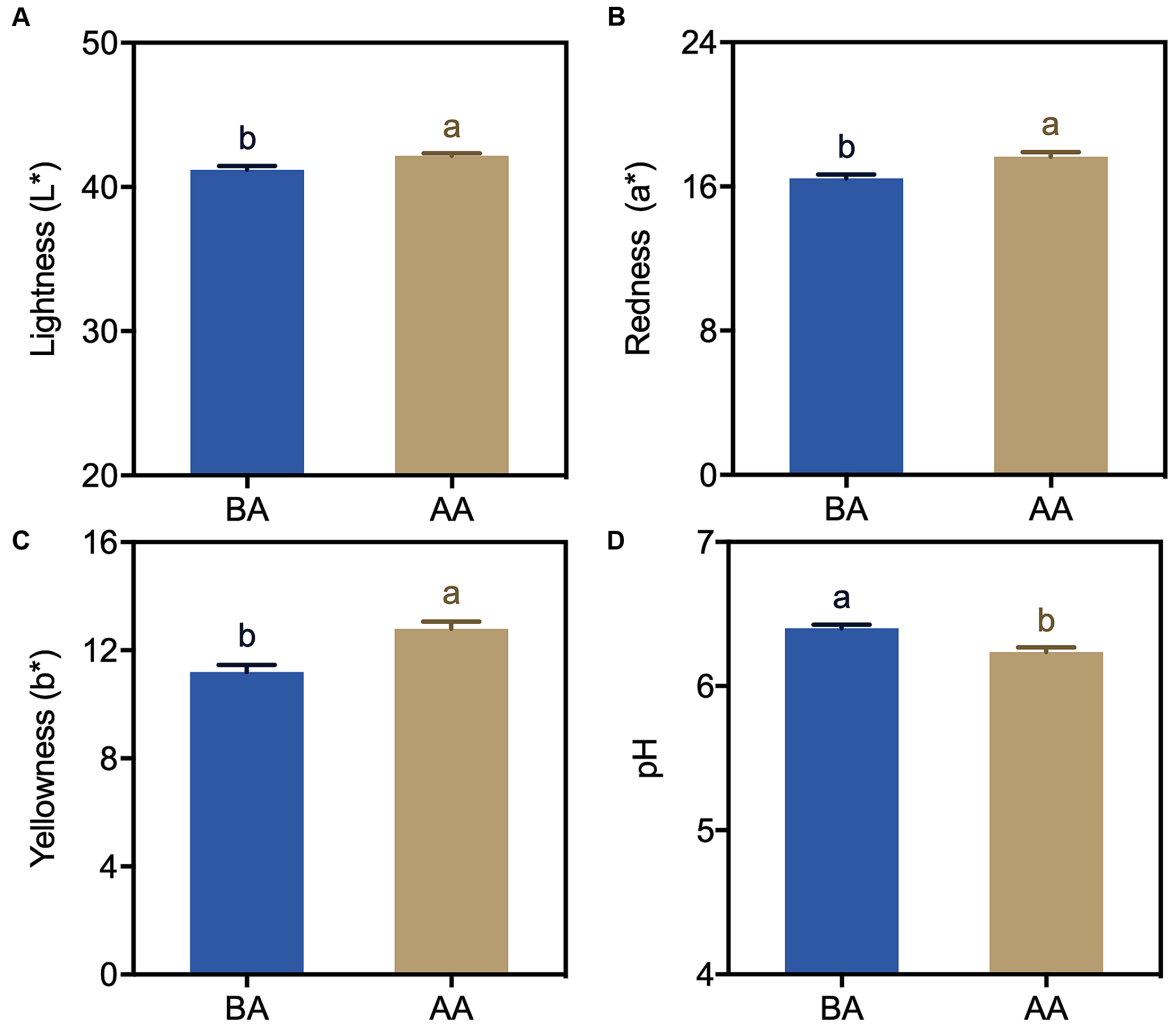
Color **(A–C)** and pH **(D)** changes in Hu sheep meat before (BA) and after the aging process (AA), with significance at *p* < 0.05 (one-way ANOVA) indicated by different lowercase letters above each column.

To investigate the impact of postmortem aging on the physicochemical properties of Hu sheep, color and pH measurements were conducted before and after the aging process (Figure 1). The results showed substantial variations in both color and pH levels between the untreated and treated samples. Notably, the aging process significantly increased the values of Lightness (L*), Redness (a*), and Yellowness (b*) in Hu sheep meat (Figures 1A–C). Compared to the non-aging-treated group, the values of L*, a* and b* were 2.4, 7.2 and 14.2% higher, respectively. Among these, the b * values changed the most. In contrast, the pH value significantly decreased as a result of the aging process (Figure 1D).

### 3.2 Effect of aging process on the mutton textural characteristics of Hu sheep

**Figure 2:**
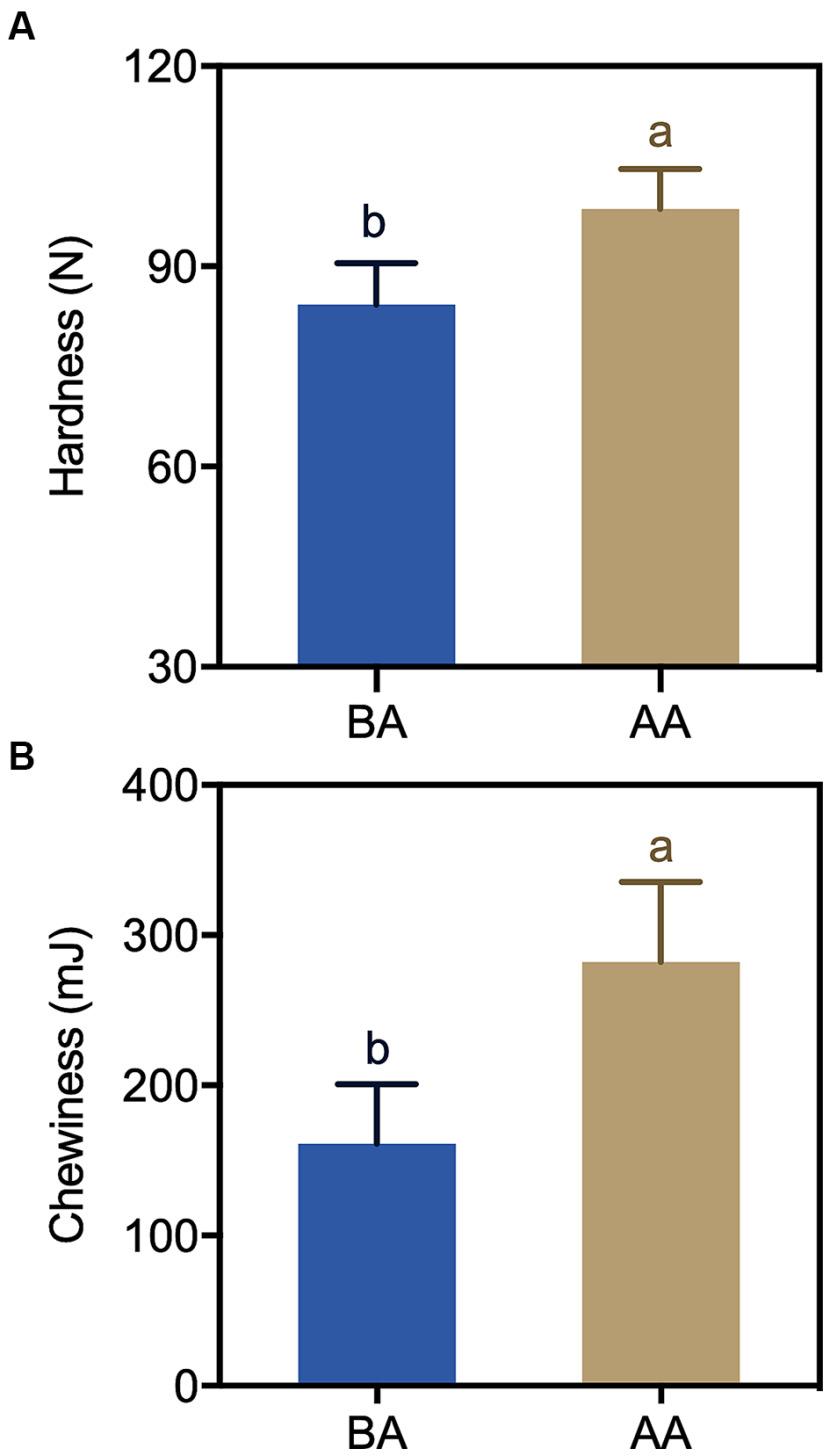
Comparison of hardness **(A)** and chewiness **(B)** of Hu sheep meat before (BA) and after the aging process (AA), with significance at *p* < 0.05 (one-way ANOVA) indicated by different lowercase letters above each column.

Textural characteristics are an important aspect in determining the sensory presentation of meat. To explore the relationship between the aging process and the textural characteristics of the meat products, the hardness and chewiness of the samples were examined. The obtained findings, as illustrated in Figure 2, demonstrated a marked elevation in the hardness of Hu sheep meat subsequent to the aging process compared to controlled samples. Furthermore, a substantial alteration in chewiness was observed under aging conditions, showing a significant surge of 75.6% relative to the controlled level.

### 3.3 Cooking loss analysis and water distribution analysis of Hu sheep meat utilizing magnetic resonance imaging and low-field nuclear MR relaxometry

**Figure 3:**
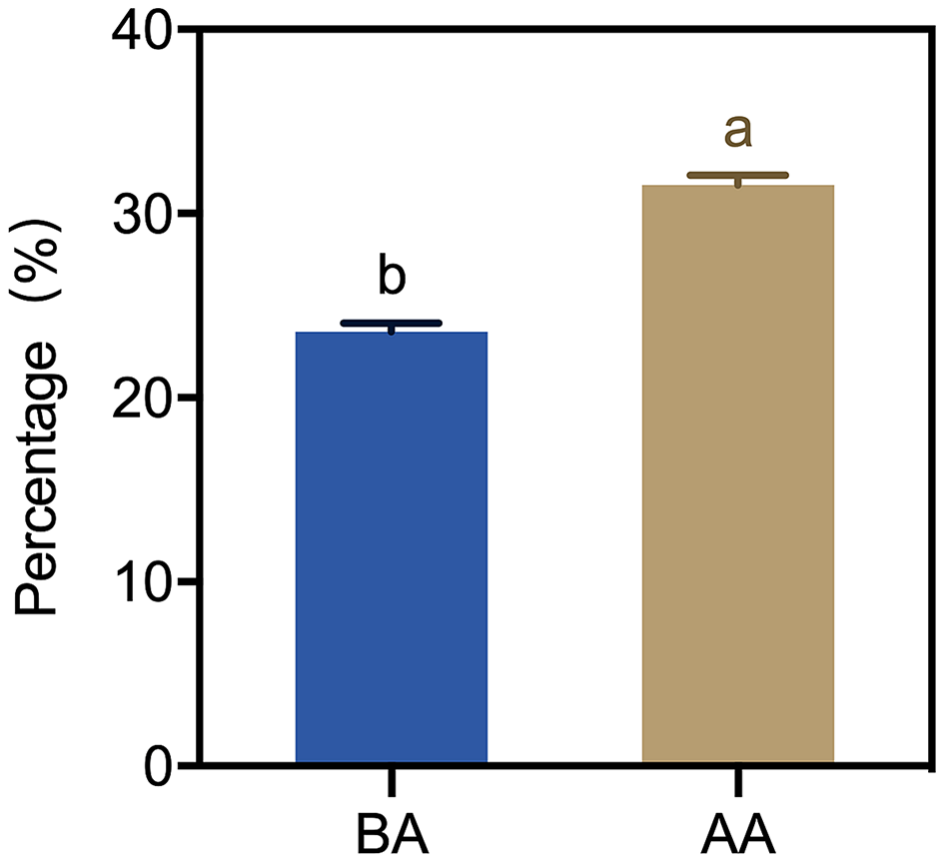
Cooking loss of Hu sheep meat before (BA) and after the aging process (AA), with significance at *p* < 0.05 (one-way ANOVA) indicated by different lowercase letters above each column.

To know the effect of the aging process on the moisture of Hu sheep meat, the cooking loss rates of meat samples were determined. A significant difference in the cooking loss of Hu sheep meat between the BA and AA group was found (Figure 3). This difference was evident in the marked increase observed after the aging process. Specifically, the loss rate of the AA group was more than 30%, which indicated that the water retention of Hu sheep meat decreased after the aging process.

**Figure 4:**
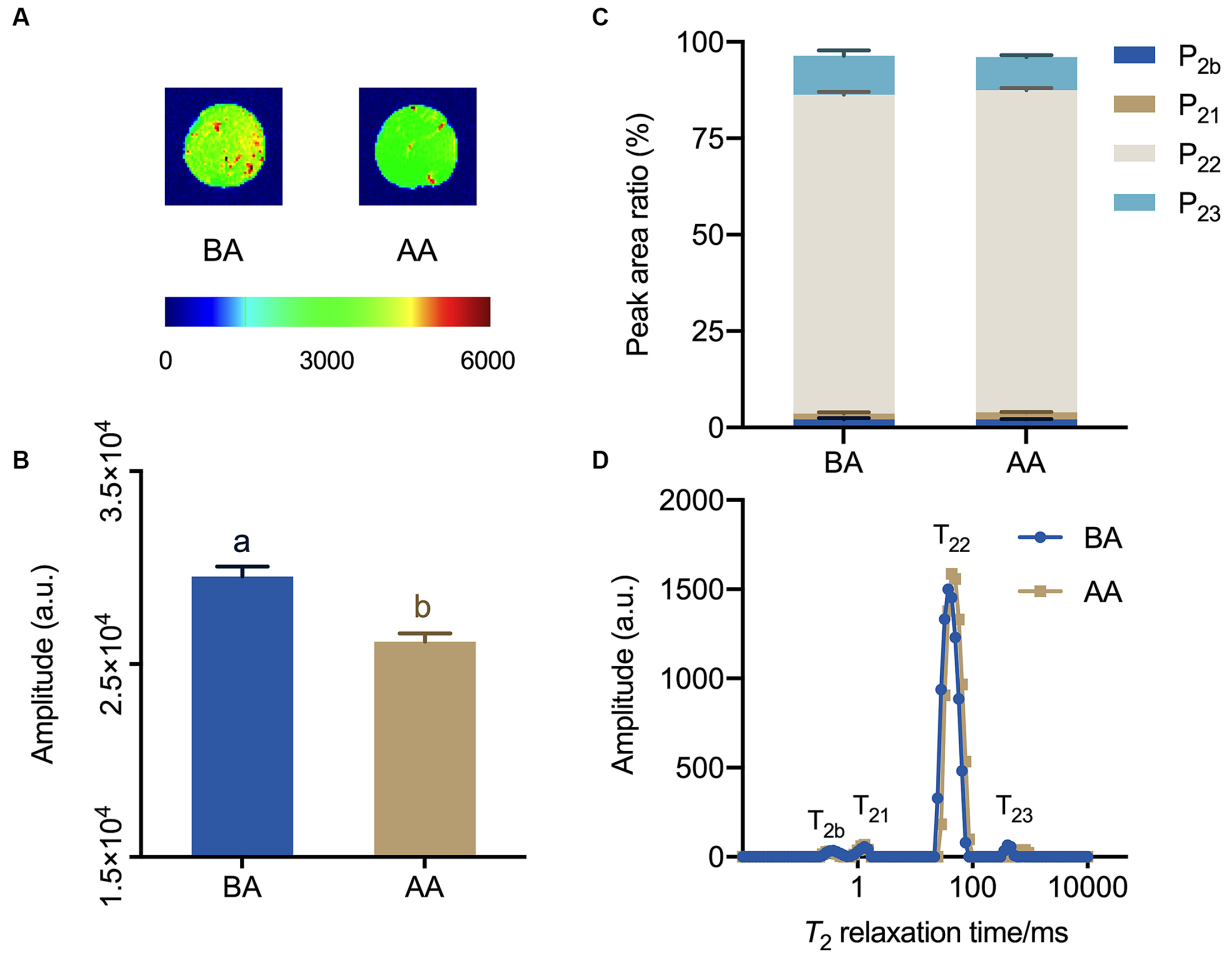
Exploration of the effects of the aging process on Hu sheep meat through magnetic resonance imaging (MRI) and Low-field nuclear MR (LF-NMR) relaxometry. **(A)** proton density-weighted images and **(B)** corresponding quantitative signal intensity histogram, **(C)** water distribution, **(D)** low-field relaxometry distributed T_2_ relaxation times, with statistically significant differences at *p* < 0.05 (one-way ANOVA) indicated by different lowercase letters above each column.

The water distribution in the BA and AA-treated meat samples was visualized using magnetic resonance imaging (MRI). By applying pseudocolor processing, the spatial distribution of water molecules in Hu sheep meat could be distinctly observed (Figure 4A). Typically, the red color in the image represents a high proton density, the blue color represents the low proton density. As shown in Figure 4A, the total signal intensity of aging Hu sheep meat decreased and the red area was unevenly distributed, with a reduction of red in the center. This suggests a decrease in water content and a change in its distribution, with water shifting from the center of the meat outward. In Figure 4B, the hydrogen proton signal intensity also significantly decreased after the aging process, indicating a reduction in water content in Hu sheep meat. This is consistent with the results observed in hydrogen proton nuclear magnetic imaging and further confirms that the aging process reduces the water holding capacity of mutton.

The analysis of multi-component relaxation characteristics revealed the presence of four distinct water populations in both BA and AA-treated mutton samples. These water populations had T_2_ relaxation times ranging from 0.1 to 1, 1 to 10, 10 to 100, and 100 to 1,000 ms, corresponding to bound water (T_2b_ and T_21_), immobilized water (T_22_), and free water (T_23_) (Figures 4C,D). In addition, the analysis showed that both BA and AA treated mutton exhibited the lowest P_2b_ and P_21_, while manifesting the highest P_22_, followed by P_23_. Consequently, immobilized water was the main water phase of mutton. Furthermore, it was observed that P_23_ exhibited a decreasing trend, indicating a significant loss of free water (Figure 4C).

### 3.4 Assembly, differentially expressed genes selection, and enrichment analysis of GO and KEGG metabolic pathways

In order to gain deeper insights into the molecular mechanism behind the aging process of the Hu sheep meat quality, subsequent transcriptomic investigations were undertaken. The sequencing and assembly results are presented in Table 1. Upon raw read filtration, a total of 40,593,128-44,781,602 clean reads were successfully obtained. The Q20 and Q30 values were calculated to be 97.46–97.72% and 93.12–93.57%, respectively. Furthermore, the GC content ranged from 44.49 to 49.98% (Table 1).

**Table 1:** Summary of assembly results of Hu sheep meat before (BA) and after aging process (AA).

Sample	Raw reads	Clean reads	Clean bases	Error (%)	Q20 (%)	Q30 (%)	GC (%)
BA1	45,553,662	44,101,392	6.62G	0.03	97.72	93.57	44.49
BA2	47,197,656	44,781,602	6.72G	0.03	97.53	93.32	46.67
BA3	41,230,828	40,593,128	6.09G	0.03	97.51	93.34	49.98
AA1	46,748,564	46,072,050	6.91G	0.03	97.59	93.48	49.09
AA2	45,088,868	44,475,286	6.67G	0.03	97.57	93.37	46.65

The Q20 and Q30 percentages indicate the proportions of nucleotides with quality values greater than 20 and 30, respectively.

**Figure 5:**
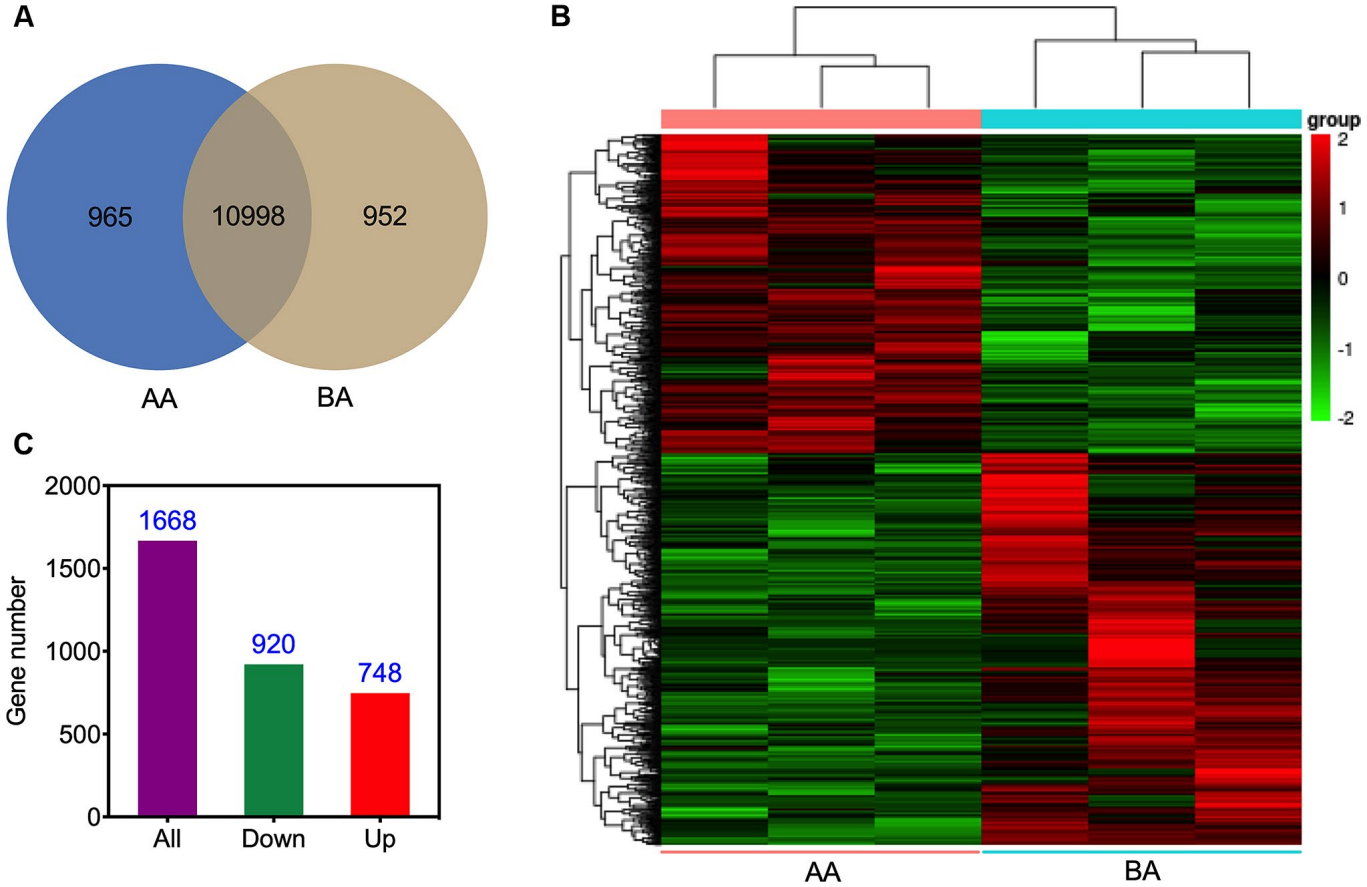
Visual representations illustrating the unigenes of Hu sheep meat before (BA) and after the aging process (AA): Venn diagram **(A)**, and hierarchical clustering **(B)**, accompanied by the histogram **(C)**, presenting the profiles of DEGs.

The transcriptomic analysis of Hu sheep yielded a comprehensive set of 12,915 genes (Figure 5A). Detailed analysis of the expression profiles of meat samples from the control and AA groups facilitated the generation of a heatmap, demonstrating the hierarchical clustering among Unigenes (Figure 5B). It is noteworthy that 10,998 genes were found to be present in both treatment groups. Moreover, specifically in the AA and BA treated groups, there were 965 and 952 genes, respectively, that were exclusively obtained. Further analysis uncovered a total of 1,668 genes exhibiting differential expression. Among these, 920 genes displayed a down-regulated pattern, while 748 genes were observed to be up-regulated (Figure 5C).

**Figure 6:**
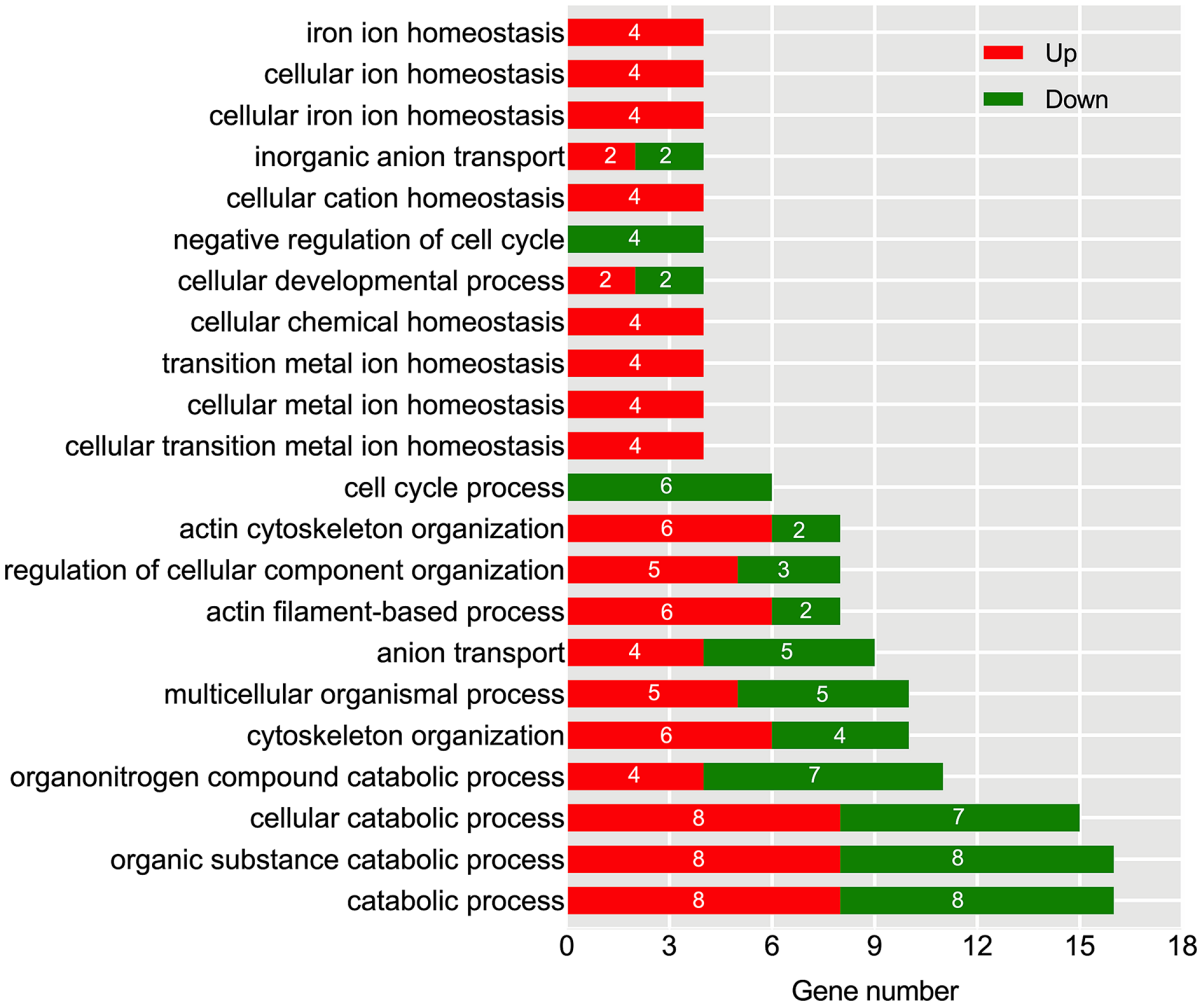
Identification of significantly enriched GO terms based on biological process after the aging process. The x-axis represents the number of DEGs, while the left y-axis displays various functional groups. Up-regulated DEGs are highlighted in red, and down-regulated DEGs are highlighted in green.

To explore the functional distribution characteristics of DEGs and their corresponding metabolic pathways, we conducted GO and KEGG enrichment analyses. The GO analysis results showed that there were 22 significantly affected biological pathways under aging conditions (Figure 6). Among them, there was a substantial enrichment of DEGs within specific biological processes, namely catabolic process, organic substance catabolic process, and cellular catabolic process. Furthermore, in the categories of iron ion homeostasis, cellular ion homeostasis, cellular iron ion homeostasis, cellular chemical homeostasis, transition metal ion homeostasis, and cellular metal ion homeostasis, only four upregulated DEGs were identified. Similarly, in the categories of negative regulation of the cell cycle and cell cycle process, only four and six down-regulated DEGs were found.

**Figure 7:**
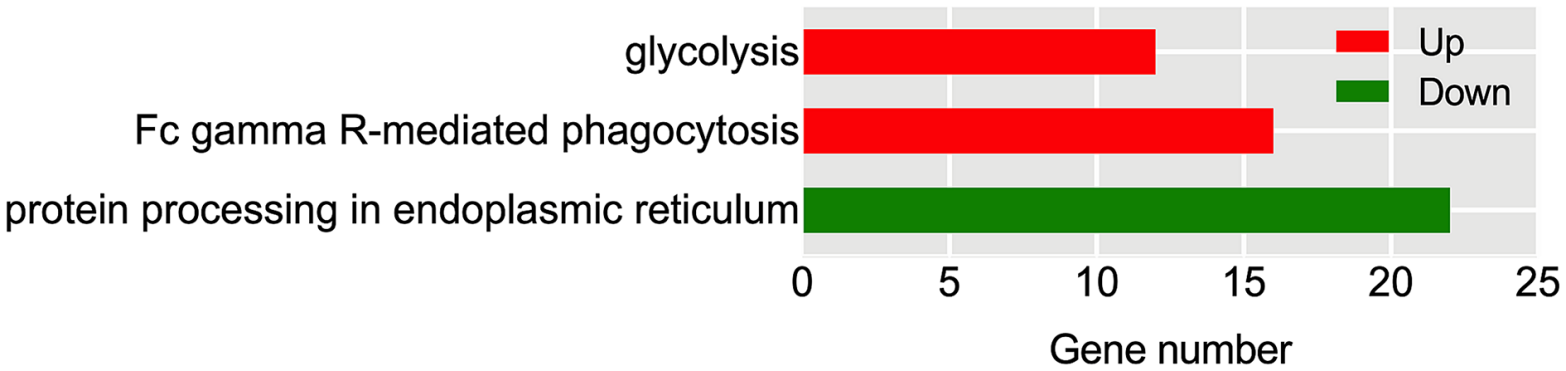
KEGG enrichment analysis of Hu sheep meat after the aging process. The x-axis represents the number of DEGs, while the left y-axis reflects the diversity of functional groups. KEGG pathways related to up-regulated DEGs are highlighted in red, and those associated with down-regulated DEGs are highlighted in green.

**Figure 8:**
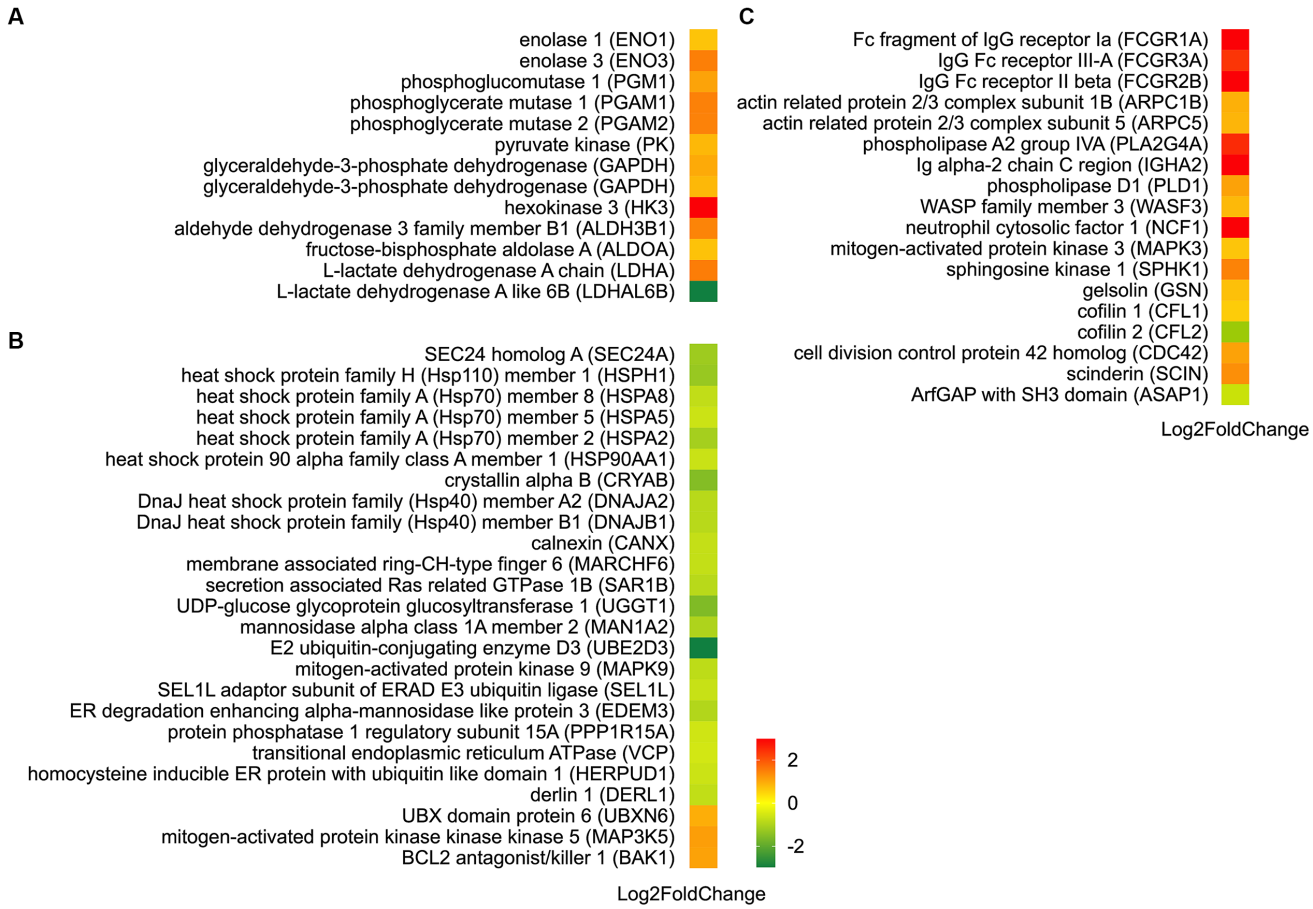
Identification of DEGs in metabolic pathways: glycolysis **(A)**, protein processing in endoplasmic reticulum **(B)**, and FcγR-mediated phagocytosis **(C)**. Up-regulated DEGs are denoted in red, whereas down-regulated DEGs are represented in green. The color bar in the lower left corner signifies the intensity of gene expression profiling.

Further investigation conducted through KEGG pathway analysis revealed a notable enrichment of up-regulated DEGs within crucial pathways (Figure 7), such as glycolysis and FcγR-mediated phagocytosis. Conversely, the down-regulated DEGs displayed a conspicuous enrichment in the protein processing in endoplasmic reticulum. Specific analysis showed that 13 DEGs were involved in the glycolysis metabolic pathway (Figure 8). Out of these, 10 DEGs were found to participate in the biosynthesis of pyruvate and exhibited an up-regulated expression. Furthermore, 2 DEGs (one up-regulated and one down-regulated) and 1 DEG (up-regulated) were discovered to participate in the subsequent synthesis of lactic acid and alcohol (Figure 8), respectively. In FcγR-mediated phagocytosis, a total of 18 DEGs were identified, with 16 up-regulated and 2 down-regulated (Figure 8). In the metabolic pathway of protein processing in the endoplasmic reticulum, a comprehensive total of 25 DEGs were identified. It is noteworthy that the majority of these DEGs exhibited a significant down-regulation, while only a mere 3 genes displayed an up-regulated pattern (Figure 8). These genes play diverse roles, to varying extents, in essential biological processes including protein synthesis and degradation, such as the ubiquitination-mediated degradation of misfolded proteins.

### 3.5 Construction and modular analysis of protein–protein interaction network

**Figure 9:**
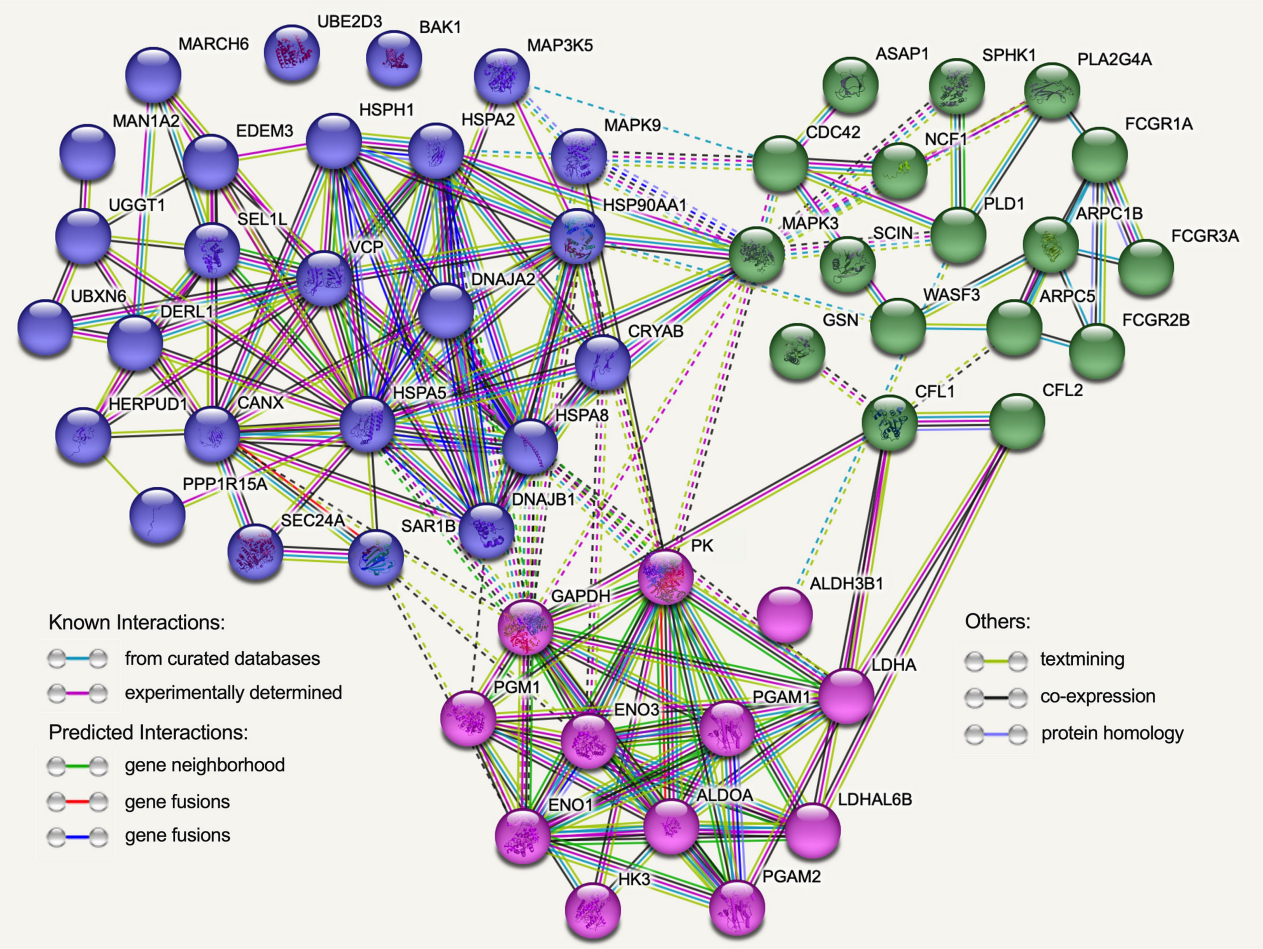
The protein–protein interaction (PPI) network was established using the functional enrichment analysis conducted on Hu sheep after the aging process. Each node in the network corresponds to DEGs encoding proteins, and the edges symbolize associations between these proteins. Blue nodes specifically represent proteins linked to protein processing in endoplasmic reticulum, while green nodes represent proteins associated with Fc gamma R-mediated phagocytosis, and red nodes indicate proteins associated with glycolysis.

By utilizing the STRING online database and the Cytoscape software, we successfully generated a PPI network in order to elucidate potential functional modules pertaining to Hu sheep meat quality after the aging process. The findings from the protein–protein interaction (PPI) analysis unveiled that, upon undergoing the aging process, a total of 54 nodes and 177 edges were acquired. Moreover, the PPI analysis discerned the existence of an intricate regulatory network interconnecting glycolysis, Fc gamma R-mediated phagocytosis, and protein metabolism throughout the course of the aging process (Figure 9; Supplementary Table S1).

**Figure 10:**
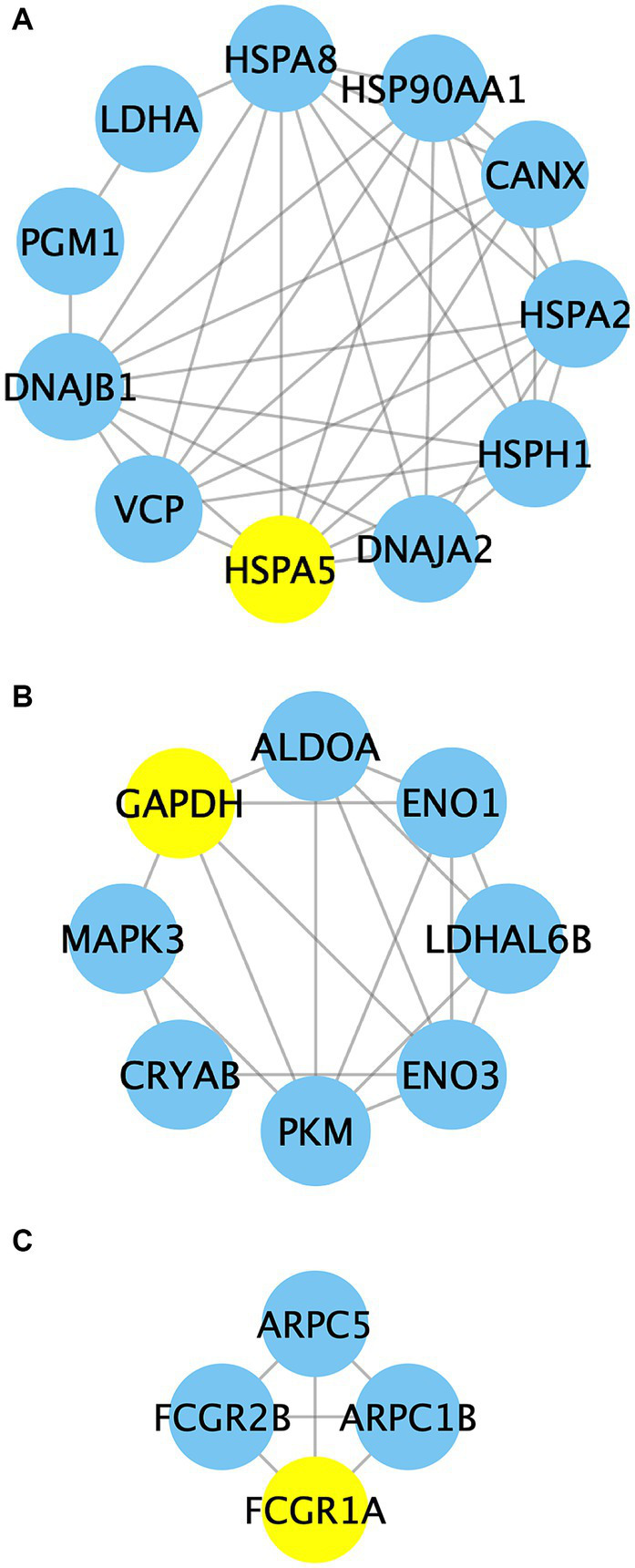
The PPI network analysis of identified proteins in clusters 1–3. Nodes in the network represent the relevant proteins, while edges symbolize the associations between them. Yellow circles highlight the predicted seed nodes within each cluster.

Additionally, the implementation of the MCODE plugin led to the identification of three distinct functional modules, as depicted in Figure 10 and Supplementary Table S2. Cluster 1 encompasses a total of 11 nodes and 37 edges, with HSPA5 serving as the seed node. The proteins within Cluster 1 primarily exhibit associations with protein processing and carbohydrate metabolism. Cluster 2 consists of 8 nodes and 18 edges, with GAPDH as the seed node, predominantly participating in glycolysis, the mitogen-activated protein kinase (MAPK) cascade, and protein metabolism. Cluster 3 comprises 4 nodes and 6 edges, with FCGR1A acting as the seed node. The proteins within this particular module mainly exhibit involvement in Fc gamma R-mediated phagocytosis.

## 4 Discussion

Hu sheep (*O. aries*) is a crucial breed in China’s efforts to conserve domesticated ovine genetic resources. It is essential in the Taihu Plain and known for its excellent qualities, such as high lamb yield, favorable wool characteristics, rapid growth, and optimal meat output (11, 12). Currently, frozen storage is the primary method for mutton preservation, as it inhibits the growth of surface microorganisms and slows down biochemical reactions, and thus plays a vital role in the circulation, sales, and storage process (15, 25). Improper freezing methods can deteriorate mutton quality by accelerating protein denaturation through prolonged freezing time and thawing, thereby reducing its edibility. It was reported that the appropriate aging process subsequent to livestock slaughter is widely recognized as a pivotal factor in the establishment of optimal meat quality (2, 4). However, due to limited genetic resources, research on Hu sheep is still at its preliminary stages. To enhance the understanding of the molecular mechanisms governing postmortem aging-induced changes in meat quality in Hu sheep, transcriptomics analysis is crucial. Our study findings elucidate the fundamental involvement of glycolysis, protein processing in endoplasmic reticulum, and the FcγR-mediated phagocytosis pathway in the determination of meat quality following postmortem aging in Hu sheep.

### 4.1 Implication of aging process in quality alterations of Hu sheep meat through glycolysis-related gene expression regulation

Appearance color is the most direct indicator for evaluating the edible quality of meat, exerting a considerable influence on consumers’ purchasing inclination (26). In this study, the color of Hu sheep meat changed during the post slaughter aging process (Figure 1). Specifically, the lightness (L*) exhibited a significant augmentation (Figure 1A), potentially attributable to the enzymatic degradation of mutton proteins leading to protein structure deterioration and enhanced light dispersion. Additionally, both the redness (a*) and yellowness (b*) values demonstrated noteworthy increments when compared to the control group (Figures 1B,C). Previous research has established the association between the a* and b* values with the oxidation–reduction state of myoglobin and lipid oxidation, respectively (27). The increased a* and b* values in this study could potentially stem from the generation of oxymyoglobin (OxyMb) through the oxygen bonding with myoglobin (Mb) under aerobic conditions, along with the accumulation of metmyoglobin (MetMb).

The pH dynamics greatly affect meat quality, impacting important aspects such as color, tenderness, and water holding capacity (28). After slaughter, animal pH levels start neutral or slightly alkaline and gradually decline. Our study found that pH significantly decreased under aging process (Figure 1D). The accumulation of lactic acid resulting from glycolysis during the early postmortem period emerged as a significant contributing factor to the decline in muscle pH levels (29). Another reason might be due to ATP depletion, resulting in a lower ATP threshold. It was also reported that the conversion between ATP and ADP, impacting muscle fiber cross-linking and meat hardening (10, 30). This explains the increased hardness and chewiness observed in the present study (Figure 2). These findings are consistent with Abdullah and Qudsieh (2)’s research, which showed a significant pH decrease within 24 h after postmortem aging in lamb meat.

Water retention in muscles and their products is vital for the juiciness of meat. In fact, it is established that meat contains approximately 75% water, serving as its principal constituent and exerting pivotal influence on both its functional attributes and textural characteristics (31). In this study, the cooking loss rate of AA-treated mutton significantly increased compared to controlled samples, which may be attributed to muscle stiffness and contraction after slaughter, as well as reduced protein space and muscle water retention. Additionally, moisture in meat predominantly occurs as bound water, free water, and immobilized water. After complex biochemical changes post-slaughter, the structures of muscles undergo alterations that impact the distribution and state of these three water types.

Research has shown that Low-field NMR relaxometry is an effective tool for quantifying water distribution and mobility, and its relationship to meat quality (31, 32). The MRI images of AA-treated mutton showed reduced brightness and quantitative signal intensity (Figures 4A,B). Moreover, T_22_ increased (Figures 4C,D), suggesting elevated flowability of immobilized water after the 24 h aging process. This enhancement contributes to the continuous improvement of mutton quality during subsequent aging.

Our transcriptome analysis confirmed the above findings, identifying 13 DEGs related to glycolysis (Figure 8). Among these genes, 12 were up-regulated, including genes encoding hexokinase 3 (HK3), phosphoglucomutase 1 (PGM1), fructose bisphophase aldolase A (ALDOA), glyceraldehyde-3-phosphate dehydrogenase (GAPDH), phosphoglycerate mutase (PGAM), enolase (ENO), pyruvate kinase (PK), aldehyde dehydrogenase 3 family member B1 (ALDH3B1), and L-lactate dehydrogenase A (LDHA). HK and PK are recognized as key enzymes in the glycolysis pathway. HK facilitates the conversion of glucose (Glu) into glu-6-phosphate (G6P). Previous research has indicated a potential correlation between HK2 and carcass traits and meat quality in pigs (33). PK catalyzes the final rate-limiting step of glycolysis, converting phosphoenolpyruvate and ADP into pyruvic acid and ATP. PK activity has been observed to be 10 times higher in pale, soft, and exudative (PSE) pork than that of normal meat (34). However, another study did not find a significant association between PK activity and meat quality traits such as muscle pH, L*, and drip loss (35). In our investigation, the upregulation of *HK* and *PK* expression after the aging process implies their possible role in meat quality formation of Hu sheep. The relationship between other enzymes in the glycolysis metabolic pathway and meat quality has also been extensively studied. Such as, PGM can reversibly catalyze the transfer of phosphate groups between the G1P and G6P. Silva et al. (36) found that phosphorylation of PGM1 in the early postmortem period speeds up the decline in pH, resulting in a longer sarcomere length. Wei et al. (37) used iTRAQ proteomics to study goat meat quality and found that LDH can be used to indicate tenderness, while GAPDH detects fat content. In non-aged beef, the brightness is positively correlated with PGM1, while the tenderness is negatively correlated with PGAM2 and annexin 2 (38). ALDHs are enzymes that facilitate the conversion of aldehydes into corresponding carboxylic acids. Gagaoua et al. (39) investigated 29 protein biomarkers using reverse phase protein arrays and identified the ALDH1A1 as a significant biomarker associated with beef tenderness and juiciness. This highlights its importance in assessing beef quality. The protein ENO3 plays a crucial role in muscle development and regeneration by catalyzing the conversion of 2-phospho-D-glycerate to phosphoenol-pyruvate. Guillemin et al. (40) revealed that ENO3 exhibits higher expression levels and more glycolytic in the muscles of steers compared to bulls. Moreover, previous studies have established a positive correlation between ENO and shear force (37, 41) as well as redness (42, 43). In this study, the up-regulated expression of these genes demonstrated enhanced glycolysis which is attributed to the respiratory arrest following slaughter and the subsequent interruption of oxygen supply in the muscles. Consequently, glycolysis becomes the dominant reaction, leading to the continuous conversion of glycogen into lactic acid. However, lactic acid produced by glycolysis cannot be transported to the liver or excluded from circulation, accumulating in muscles and causing a decrease in meat pH (37). These pH decline dynamics after slaughter remarkably impact meat quality. The rate of pH decrease determines the occurrence of PSE meat, while the extent of decline affects meat tenderness, water retention, color, and cooking loss (37, 44).

In summary, the 24 h aging process can up-regulate the expression of genes related to glycolysis metabolism, such as *HK*, *PGM1*, *ALDOA*, *GAPDH*, *PGAM*, *ENO*, *PK*, and *ALDH*, regulating the changes in pH, color, hardness, chewiness, and water content, thereby affecting the meat quality of Hu sheep.

### 4.2 Aging process influences endoplasmic reticulum pathway-associated gene expression for Hu sheep meat quality regulation

The endoplasmic reticulum (ER) serves as the site for synthesizing almost all lipids, secreted proteins, and transmembrane proteins. Initially, intracellular protein synthesis begins with free ribosomes in the cytoplasm. Some proteins then move to the ER membrane for further extension to complete protein synthesis. In this study, a total of 25 DEGs associated with the protein processing in the endoplasmic reticulum pathway were identified (Figure 8). Notably, calnexin (CANX), mannosidase alpha class 1A member 2 (MAN1A2), and UDP glucose glycoprotein glucose transfer 1 (UGGT1) are involved in the folding of newly synthesized glycoproteins. Secretion associated Ras related GTPase 1B (SAR1B) and SEC24 homolog A (SEC24A) play roles in the assembly and transport of COPII-coated vesicles. Research has demonstrated the importance of CANX as an ER molecular chaperone for glycoprotein folding and modification, crucial for maintaining correct folding and reducing misfolded protein accumulation (45). UGGT is a key participant in ER quality control, recognizing nearly folded proteins lacking glucose in N-oligosaccharide and catalyzing reglycosylation using UDP-Glu as a substrate. Additionally, glucosidase II removes the Glu residue. The glycosylation and deglycosylation cycle continues until the glycoproteins are correctly folded or targeted for degradation (46). SAR1, a samll GTPase, acts as a molecular switch regulating selective substance transport from ER to Golgi via SAR1-GDP/SAR1-GTP formation, playing a vital role in COPII-coated vesicle mediated protein transportation (47). Down-regulation of *CANX*, *MAN1A2*, *UGGT1*, *SAR1B*, and *SEC24A* indicates the impact of aging process on protein assembly, COPII-coated vesicle assembly and transport, ultimately influencing protein synthesis.

Abnormal protein synthesis can trigger a cascade of reactions, including dysfunction of the ER and an imbalance in calcium levels. This leads to the accumulation of unfolded or misfolded proteins in the ER, activating signaling pathways and causing ER stress (ERS) response. Three genes, protein phosphatase 1 regulatory subunit 15A (PPP1R15A), mitogen-activated protein kinase kinase 5 (MAP3K5, ASK1) and mitogen-activated protein kinase 9 (MAPK9, JNK2) (Figure 8), were found to be involved in the ERS process. The role of ER stress sensor protein kinase (PERK) in ER stress is well-established. Activation of the PERK pathway leads to phosphorylation of the eukaryotic translation initiation factor 2a (eIF2a), which inhibits protein synthesis and triggers apoptosis. Phosphorylated eIF2a selectively translates activating transcription factor 4 (ATF4), resulting in the expression of downstream proteins like GADD34. GADD34, in turn, dephosphorylates eIF2a, restoring protein synthesis and preventing inhibition (48). This study found that the expression of the *PPP1R15A* gene, which encodes GADD34, was down-regulated, suggesting that the 24 h aging process can regulate protein translation initiation through the PERK signaling pathway, leading to protein synthesis inhibition. ASK1 and JNK have been implicated in the unfolded protein response (UPR). Overexpressing *ASK1* induces apoptosis through mitochondrial-dependent caspase activation (49), whereas deletion of *ASK1* in mice inhibits NF and H_2_O_2_-induced apoptosis in ASK1^−/−^ cells (50). ASK1 can activate JNK, which impedes the anti-apoptotic function of BCL2. Deactivating BCL2 can activate BAX/BAK-mediated apoptosis (51). In this study, *BAK1*, *ASK1* and *JNK2* were differentially expressed in AA-treated mutton as compared to BA (Figure 8). This finding indicates that the 24 h aging process mainly modulates apoptosis through regulating *ASK1* and *BAK1*. Under severe stress and unrecovered ER function, cells undergo programmed cell death (52). Moreover, the ER-associated protein degradation (ERAD) pathway regulates the transport of misfolded proteins from the ER for degradation through the ubiquitin proteasome system. This study identified 16 DEGs involved in ERAD, associated with processes like substrate recognition, ubiquitination, and retrograde transport (Figure 8). These findings suggest that the 24 h aging process can potentially impact ERAD function and be linked to post-mortem metabolic disorders, such as considerable inhibition of ATP-producing aerobic metabolism.

Taken together, the 24 h aging process can affect protein synthesis, ERS response, and ERAD pathway by down-regulating the proteins processed in endoplasmic reticulum, thus having an impact on the meat quality of Hu sheep.

### 4.3 FcγR-mediated phagocytosis-associated genes were involved in Hu sheep meat quality changes after aging process

Phagocytosis is a vital mechanism for host immune defense, providing a direct route for digesting external substances. Fcγ-receptors (FcγRs), categorized as activating or inhibitory receptors, transmit appropriate signals through immunoreceptor tyrosine-based activation motifs or inhibitory motifs (53). FcγR-mediated phagocytosis involves phagocytic cup formation through actin cytoskeletal rearrangements, engulfment of particles, and release of proinflammatory mediators like cytokines and reactive oxygen species (ROS). This process is tightly regulated by activating and inhibitory FcγRs and intracellular signaling molecules (54). In this study, three FcγR genes were identified, including two genes encoding activating receptor proteins: Fc fragment of IgG receptor Ia (FCGR1A) and IgG Fc receptor III-A (FCGR3A); and one gene encoding an inhibitory receptor protein: IgG Fc receptor II beta (FCGR2B) (Figure 8). The increased expression of *FCGR1A* and *FCGR2B* signifies the activation of the FcγR-mediated phagocytosis metabolic pathway, suggesting a potential mechanism by which the aging process influences immune response and cellular clearance.

In addition, the transcriptome analysis revealed the presence of 14 additional genes inked to FcγR-mediated phagocytosis (Figure 8), and their functions have been investigated. For instance, studies have demonstrated that PLD catalyzes the hydrolysis of phosphatidylcholine, generating the lipid second messenger phosphatidic acid (PA) and choline (55). PLD is involved in various cellular functions, including intracellular protein transport and cell cytoskeleton dynamics (55, 56). SPHK, a member of the DAG kinase family, has been well characterized for its activity and function in animals and yeast. In mammals, both SPHK and its product sphingosine-1-phosphate (S1P) play essential roles in regulating numerous cellular processes (57). NCF1 (p47^phox^) is a vital NADPH oxidase subunit that, upon appropriate stimulation, assembles protein subunits, leading to ROS generation. These ROS, in turn, initiate crucial intracellular signals that govern the cell’s response to functional effects including phagocytosis (58). Extracellular signal-regulated kinases 1 and 2 (ERK1/2) are members of the mitogen-activated protein kinase (MAPK) family, involved in signal cascades and transmitting extracellular signals to intracellular targets. Research has indicated that the ERK cascade reaction involves several kinases in the MAP3K layer, including Ras/Raf/MAPK (MEK) 1/2 in the MAPKK layer, ERK1/2 (MAPK3/1) in the MAPK layer, and seveal MAPKAPKs within PLA2 in the subsequent layer. The highly regulated ERK cascade is responsible for fundamental cellular processes (59). This study suggests that the aging process could affect the cellular signaling transduction network through ROS-mediated signal transduction and the ERK cascade, as indicated by the up-regulation of *NCF1*, *MAPK3* and *PLA2G4A* genes. Cell cytoskeleton rearrangement is a well-known phenomenon that occurs in various cellular activities, enabling the transition between gel and sol states of the cytoplasm. This reversible process involves multiple proteins, including gelsolin (GSN), a crucial member of the gelsolin superfamily. GSN binds to actin and regulates its polymerization and depolymerization, playing a significant role in actin dynamics, cell movement, apoptosis, and phagocytosis (60). Another member of this family, scinderin (SCIN), shows the closest similarity to GSN. SCIN is found in all secretory cells and participates in the remodeling of the actin cytoskeleton during secretion processes (60, 61). Cdc42, discovered in yeast, belongs to the Rho subfamily of small GTPases. It acts as a potent regulator of actin cytoskeleton dynamics, cell adhesion interactions, and motility, while also playing vital roles in gene expression, proliferation, and apoptosis (62). In addition, studies have shown that Arp2/3 nucleates branched actin filaments and is important for cell motility, endocytosis, and phagocytosis. This activity is stimulated by nucleation promoting factors including Wiskott-Aldrich syndrome protein (WASP) and ASP family verprolin-homologous protein (WAVE) (63). The WAVE family proteins have the function of regulating the actin cytoskeleton (64). In our study, we observed an up-regulation of *GSN*, *SCIN*, *CDC42*, *ARPC1B*, *ARPC5*, and *WASF3* (*WAVE3*), implying an important role of cytoskeleton remodeling in the quality formation of Hu sheep meat.

Collectively, the 24 h aging process can modulate the immune response, cellular clearance, and cell cytoskeleton rearrangement by enhancing the expression of FcγR-mediated phagocytosis-associated genes, ultimately impacting the meat quality of Hu sheep.

### 4.4 Protein–protein interaction network

Protein complexes in cells play crucial roles in various cellular processes (65, 66). PPI analysis is an influential approach for investigating the intricate functionality of proteins and their networks at a network-based level (65). In this study, the interaction networks of DEGs were analyzed and visualized using the STRING online database and Cytoscape software. Subsequently, the MCODE plugin was applied to identify potential functional modules within the network, leading to the discovery of three clusters (Figure 10; Supplementary Table S2). In cluster 1, there are 11 nodes and 37 edges that are primarily associated with protein metabolism and glycolysis. Among them, 70 kDa heat shock protein 5 (HSPA5) serves as the seed node. HSPA5 is located within the ER lumen and plays a dual role as a typical HSP70 chaperone. It assists in the folding and assembly of proteins while also acting as a key regulator of ER homeostasis (67). Previous studies have investigated the correlation between HSPs and meat quality. Zhang et al. (68) observed decreased levels of HSP90 in the *Longissimus dorsi* muscle of pigs with low pH and discovered a significant negative correlation between HSP90 levels and cooking loss, drip loss, and brightness. Sanchez, et al. (67) proposed HSPA5 as a potential biomarker for heat stress in Guang Ming Broilers. Laville et al. (69) found the absence of HSP27 in samples of the PSE zones in pig semimembranosus muscle compared to the normal treatment group. Our analysis indicates that the main interaction involving HSPA5 and the nearby proteins was obtained from curated databases, suggesting that HSPA5 might function as a master regulator in this sub-network. In cluster 2, GAPDH, ALDOA, ENO1, ENO3, LDHAL6B, and PKM participate in glycolytic metabolism. MAPK3 is involved in the MAPK signaling pathway, while CRYAB acts as a molecular chaperone in protein metabolism. This suggests a close connection between glycolysis, the MAPK signaling pathway, and protein metabolism. Additionally, GAPDH acts as the seed node in this cluster. It has been reported that GAPDH is a key enzyme that converts 3-phosphoglycerate (3-PGA) to glyceraldehyde 3-phosphate (G3P) (70). Along with other endogenous enzyme systems, GAPDH is believed to play important roles in postmortem protein hydrolysis and meat tenderization (71). A recent study revealed that GAPDH, ATP-dependent 6-phosphofructokinase (PFKM), and PKM may directly interact with other differentially expressed proteins, affecting glycolytic muscle characteristics (72). The interactions among these proteins highlight the vital role of GAPDH in interacting with adjacent proteins like PKM, and thus participating in regulating the quality changes in mutton. Cluster 3 proteins are primarily engaged in FcγR-mediated phagocytosis, with FCGR1A being predicted as the seed node. FcγRs play a vital role in both humoral and cellular immune responses due to their interaction with the Fc region of IgG (53). FCGR1A is the only high-affinity receptor for IgG and functions in both innate and adaptive immune responses. In this study, FCGR1A may participate in the immune response and contribute to the quality maintenance of mutton in Hu sheep through its interaction with adjacent proteins. Taken together, PPI analysis reveals an intricate regulatory network connecting glycolysis, the MAPK signaling pathway, protein metabolism, and immune response during the aging process in Hu sheep meat. Nevertheless, further investigation is warranted to elucidate the specific regulatory mechanisms involved.

## 5 Conclusion

**Figure 11:**
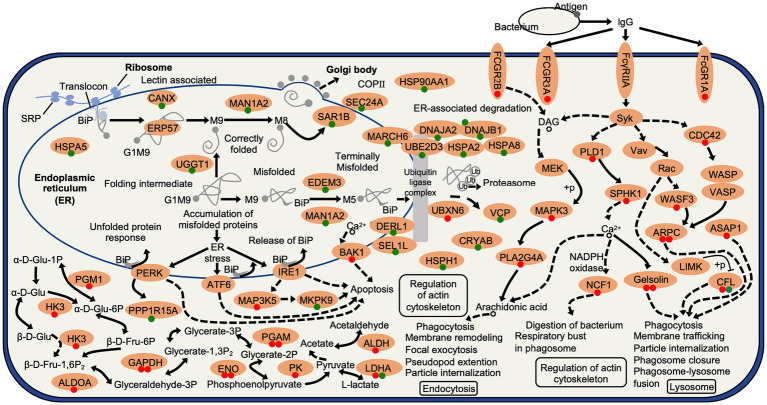
Schematic presentation of the aging process effects on Hu sheep meat. The expression profiles of DEGs are highlighted and denoted by circles, with red and green colors signifying up- and down-regulated expression levels. ALDH, aldehyde dehydrogenase; ALDOA, fructose-bisphosphate aldolase A; ARPC, actin related protein 2/3 complex; ASAP1, ArfGAP with SH3 domain; ATF6, activating transcription factor 6; BAK1, BCL2 antagonist/killer 1; CANX, calnexin; CDC42, cell division control protein 42 homolog; CFL, cofilin; CRYAB, crystallin alpha B; DAG, Diacylglycerol; DERL1, derlin 1; α-D-Glu-1-P, α-D-Glucose-1-phosphate; β-D-Fru-1,6P_2_, β-D-Fructose-1,6-diphosphate; DNAJA2, DnaJ heat shock protein family (Hsp40) member A2; DNAJB1, DnaJ heat shock protein family (Hsp40) member B1; ENO, enolase; ERP57, protein disulfide-isomerase A3; FCGR1A, Fc fragment of IgG receptor Ia; FCGR3A, IgG Fc receptor III-A; FCGR2B, IgG Fc receptor II beta; FcγRIIA, low affinity immunoglobulin gamma Fc region receptor II-A; GAPDH, glyceraldehyde-3-phosphate dehydrogenase; Glyceraldehyde-3P, glyceraldehyde 3-phosphate; Glycerate-1,3P_2_, glycerate-1,3-bisphosphate; HK3, hexokinase 3; HSP, heat shock protein; IgG, Immunoglobulin G; IRE1, inositol-requiring enzyme-1; MAN1A2, mannosidase alpha class 1A member 2; MAP3K5, mitogen-activated protein kinase kinase kinase 5; MAPK9, mitogen-activated protein kinase 9; MEK, mitogen-activated protein kinase kinase 1; NCF1, neutrophil cytosolic factor 1; LDHA, L-lactate dehydrogenase A; LIMK, LIM domain kinase 1; PERK, PKR-like endoplasmic reticulum kinase; PGAM, phosphoglycerate mutase; PGM, phosphoglucomutase; PK, pyruvate kinase; PLD1, phospholipase D1; PLA2G4A, phospholipase A2 group IVA; PPPIR15A, protein phosphatase 1 regulatory subunit 15A; Rac, Ras-related C3 botulinum toxin substrate 1; SAR1B, secretion associated Ras related GTPase 1B; SEC24A, SEC24 homolog A; SEL1L, SEL1L adaptor subunit of ERAD E3 ubiquitin ligase; SRP, signal recognition particle; Syk, spleen tyrosine kinase; UBE2D3, E2 ubiquitin-conjugating enzyme D3; VASP, vasodilator-stimulated phosphoprotein; Vav, guanine nucleotide exchange factor VAV; VCP, transitional endoplasmic reticulum ATPase; WASF3, WASP family member 3; WASP, Wiskott-Aldrich syndrome protein.

This study was conducted with the aim of investigating the impacts of a 24 h aging process on the physiological and transcriptomic changes of Hu sheep meat. The results obtained from our study demonstrate that the application of the aging process leads to significant increases in the L*, a*, b* values, as well as its hardness and chewiness, while concurrently causing a notable decrease in pH value. Furthermore, the aging process has a discernible influence on the water content. Through transcriptomic analysis, it has been revealed that the primary effects of the 24 h aging process predominantly involve the modulation of glycolysis metabolism, protein processing in endoplasmic reticulum, and the FcγR-mediated phagocytosis pathway, thereby facilitating changes in the mutton quality attributes. In light of these discoveries, a schematic diagram has been devised to visually depict the observed 24 h aging process effects on Hu sheep (Figure 11).

## Data availability statement

The supporting data is publicly available in NCBI SRA with the accession numbers SRR25065590-SRR25065595.

## Ethics statement

The animal study was approved by Animal Ethics Committee of the Bengbu University. The study was conducted in accordance with the local legislation and institutional requirements.

## Author contributions

HL: Conceptualization, Methodology, Software, Data curation, Formal analysis, Funding acquisition, Investigation, Project administration, Resources, Supervision, Visualization, Writing – original draft, Writing – review & editing. Y-HF: Methodology, Writing – review & editing, Conceptualization, Software, Supervision. CX: Supervision, Validation, Writing – review & editing. YC: Supervision, Validation, Writing – review & editing. X-YL: Supervision, Validation, Writing – review & editing. YW: Supervision, Validation, Writing – review & editing. L-LQ: Writing – original draft, Validation. M-YZ: Validation, Supervision, Writing – review & editing. G-YG: Supervision, Validation, Writing – review & editing. Y-FM: Writing – review & editing, Supervision, Validation. Y-LY: Writing – review & editing, Supervision, Validation. QT: Writing – review & editing, Supervision. K-QL: Writing – review & editing, Supervision, Validation. Y-TL: Writing – review & editing, Supervision, Validation. C-TL: Writing – review & editing, Supervision, Validation. X-YR: Writing – review & editing, Conceptualization, Funding acquisition, Methodology, Project administration, Software, Supervision. M-YW: Data curation, Formal analysis, Investigation, Resources, Visualization, Writing – original draft, Writing – review & editing, Conceptualization, Funding acquisition, Methodology, Project administration, Software, Supervision. BZ: Conceptualization, Funding acquisition, Methodology, Project administration, Software, Supervision, Writing – review & editing.

## Footnotes

^1^https://magic.novogene.com
