# Supplementary material for: Physiological and transcriptomic analysis dissects the molecular mechanism governing meat quality during postmortem aging in Hu sheep (Ovis aries)
Source: Front Nutr. 2024 Jan 5;10:1321938. doi: 10.3389/fnut.2023.1321938 (PMC10799347; doi:10.3389/fnut.2023.1321938)
Supplement: Supplementary file 1 [file Table_1.doc]

**Table S1.** The detailed information of interaction network of DEGs generated by STRING 12.0 database.

| Node1 | Node2 | Neighborhood  on  chromosome | Phylogenetic cooccurrence | Homology | Coexpression | Experimentally  determined interaction | Database annotated | Automated textmining | Combined  score |
| --- | --- | --- | --- | --- | --- | --- | --- | --- | --- |
| ALDH3B1 | PLD1 | 0 | 0 | 0 | 0 | 0 | 0.462 | 0.042 | 0.462 |
| ALDOA | LDHAL6B | 0 | 0 | 0 | 0.111 | 0.136 | 0.6 | 0.333 | 0.767 |
| ALDOA | HK3 | 0 | 0 | 0 | 0.066 | 0 | 0.6 | 0.287 | 0.71 |
| ALDOA | PGAM1 | 0.104 | 0 | 0 | 0.158 | 0.12 | 0.8 | 0.236 | 0.88 |
| ALDOA | LDHA | 0 | 0 | 0 | 0.114 | 0.136 | 0.6 | 0.66 | 0.882 |
| ALDOA | ENO3 | 0 | 0 | 0 | 0.407 | 0.22 | 0.659 | 0.454 | 0.902 |
| ALDOA | PGAM2 | 0.104 | 0 | 0 | 0.158 | 0.176 | 0.8 | 0.435 | 0.916 |
| ALDOA | PGM1 | 0 | 0 | 0 | 0.298 | 0.045 | 0.851 | 0.287 | 0.919 |
| ALDOA | ENO1 | 0 | 0 | 0 | 0.407 | 0.22 | 0.659 | 0.554 | 0.92 |
| ALDOA | PK | 0.121 | 0 | 0 | 0.275 | 0.106 | 0.8 | 0.608 | 0.947 |
| ALDOA | GAPDH | 0.107 | 0 | 0 | 0.554 | 0.367 | 0.98 | 0.416 | 0.996 |
| ARPC1B | ARPC5 | 0 | 0 | 0 | 0.61 | 0.956 | 0.807 | 0.681 | 0.998 |
| ARPC1B | FCGR3A | 0 | 0 | 0 | 0.063 | 0 | 0.604 | 0 | 0.613 |
| ARPC1B | FCGR2B | 0 | 0 | 0 | 0.061 | 0 | 0.541 | 0 | 0.55 |
| ARPC1B | WASF3 | 0 | 0 | 0 | 0.059 | 0 | 0.538 | 0.167 | 0.606 |
| ARPC1B | FCGR1A | 0 | 0 | 0 | 0.062 | 0 | 0.6 | 0 | 0.608 |
| ARPC5 | CFL1 | 0 | 0 | 0 | 0.068 | 0 | 0 | 0.501 | 0.515 |
| ARPC5 | FCGR2B | 0 | 0 | 0 | 0.06 | 0 | 0.541 | 0 | 0.55 |
| ARPC5 | FCGR1A | 0 | 0 | 0 | 0.059 | 0 | 0.6 | 0 | 0.607 |
| ARPC5 | WASF3 | 0 | 0 | 0 | 0 | 0 | 0.538 | 0.234 | 0.63 |
| ARPC5 | ARPC1B | 0 | 0 | 0 | 0.61 | 0.956 | 0.807 | 0.681 | 0.998 |
| ASAP1 | CDC42 | 0 | 0 | 0 | 0 | 0.136 | 0.251 | 0.29 | 0.5 |
| CANX | HSPA8 | 0 | 0 | 0 | 0.115 | 0.198 | 0.074 | 0.35 | 0.515 |
| CANX | HSPA2 | 0 | 0 | 0 | 0.115 | 0.198 | 0.074 | 0.246 | 0.438 |
| CANX | EDEM3 | 0 | 0 | 0 | 0.063 | 0.484 | 0 | 0.639 | 0.81 |
| CANX | SEL1L | 0 | 0 | 0 | 0.072 | 0.041 | 0 | 0.643 | 0.654 |
| CANX | DNAJB1 | 0 | 0 | 0 | 0.069 | 0.147 | 0 | 0.331 | 0.422 |
| CANX | HSPH1 | 0 | 0 | 0 | 0.116 | 0.198 | 0.074 | 0.256 | 0.446 |
| CANX | SAR1B | 0 | 0 | 0 | 0.067 | 0 | 0.411 | 0.205 | 0.524 |
| CANX | GAPDH | 0 | 0 | 0 | 0.059 | 0 | 0 | 0.523 | 0.532 |
| CANX | HSP90AA1 | 0 | 0 | 0 | 0.225 | 0.153 | 0 | 0.43 | 0.593 |
| CANX | DERL1 | 0 | 0 | 0 | 0.088 | 0.05 | 0 | 0.584 | 0.608 |
| CANX | SEC24A | 0 | 0 | 0 | 0.157 | 0.045 | 0.363 | 0.429 | 0.667 |
| CANX | UGGT1 | 0 | 0 | 0 | 0.142 | 0.342 | 0 | 0.69 | 0.809 |
| CANX | VCP | 0 | 0 | 0 | 0.091 | 0.776 | 0 | 0.58 | 0.907 |
| CANX | HSPA5 | 0 | 0 | 0 | 0.724 | 0.688 | 0.311 | 0.835 | 0.988 |
| CDC42 | MAP3K5 | 0 | 0 | 0 | 0 | 0 | 0.8 | 0 | 0.8 |
| CDC42 | MAPK9 | 0 | 0 | 0 | 0.059 | 0.205 | 0.849 | 0.057 | 0.879 |
| CDC42 | MAPK3 | 0 | 0 | 0 | 0 | 0.203 | 0.202 | 0.246 | 0.479 |
| CDC42 | ASAP1 | 0 | 0 | 0 | 0 | 0.136 | 0.251 | 0.29 | 0.5 |
| CDC42 | PLD1 | 0 | 0 | 0 | 0 | 0.148 | 0.31 | 0.149 | 0.456 |
| CDC42 | NCF1 | 0 | 0 | 0 | 0.059 | 0.316 | 0.199 | 0.147 | 0.501 |
| CDC42 | WASF3 | 0 | 0 | 0 | 0 | 0.129 | 0.275 | 0.424 | 0.604 |
| CFL1 | ARPC5 | 0 | 0 | 0 | 0.068 | 0 | 0 | 0.501 | 0.515 |
| CFL1 | LDHAL6B | 0 | 0 | 0 | 0.067 | 0.393 | 0 | 0.047 | 0.413 |
| CFL1 | GSN | 0 | 0 | 0 | 0.06 | 0.051 | 0 | 0.612 | 0.623 |
| CFL1 | CFL2 | 0 | 0 | 0.978 | 0.047 | 0.196 | 0.8 | 0.655 | 0.835 |
| CFL1 | GAPDH | 0 | 0 | 0 | 0.14 | 0.09 | 0 | 0.325 | 0.425 |
| CFL1 | LDHA | 0 | 0 | 0 | 0.067 | 0.393 | 0 | 0.122 | 0.459 |
| CFL2 | LDHAL6B | 0 | 0 | 0 | 0.067 | 0.393 | 0 | 0.047 | 0.413 |
| CFL2 | LDHA | 0 | 0 | 0 | 0.067 | 0.393 | 0 | 0.073 | 0.429 |
| CFL2 | CFL1 | 0 | 0 | 0.978 | 0.047 | 0.196 | 0.8 | 0.655 | 0.835 |
| CRYAB | HSPA8 | 0 | 0 | 0 | 0.069 | 0.15 | 0.513 | 0.339 | 0.711 |
| CRYAB | HSPA2 | 0 | 0 | 0 | 0.069 | 0.15 | 0.431 | 0.205 | 0.594 |
| CRYAB | MAPK3 | 0 | 0 | 0 | 0 | 0.149 | 0.272 | 0.11 | 0.4 |
| CRYAB | HSP90AA1 | 0 | 0 | 0 | 0.067 | 0.155 | 0.523 | 0.457 | 0.768 |
| CRYAB | DNAJB1 | 0 | 0 | 0 | 0.073 | 0.071 | 0.516 | 0.414 | 0.723 |
| CRYAB | ENO3 | 0 | 0 | 0 | 0.064 | 0.047 | 0 | 0.389 | 0.407 |
| CRYAB | HSPA5 | 0 | 0 | 0 | 0.053 | 0.093 | 0.137 | 0.332 | 0.438 |
| DERL1 | SEL1L | 0 | 0 | 0 | 0.087 | 0.495 | 0.603 | 0.832 | 0.965 |
| DERL1 | CANX | 0 | 0 | 0 | 0.088 | 0.05 | 0 | 0.584 | 0.608 |
| DERL1 | UBXN6 | 0 | 0 | 0 | 0.059 | 0.103 | 0 | 0.352 | 0.405 |
| DERL1 | HERPUD1 | 0 | 0 | 0 | 0 | 0.123 | 0 | 0.485 | 0.529 |
| DERL1 | HSPA5 | 0 | 0 | 0 | 0.099 | 0.077 | 0 | 0.489 | 0.538 |
| DERL1 | MARCH6 | 0 | 0 | 0 | 0 | 0.072 | 0.113 | 0.508 | 0.559 |
| DERL1 | VCP | 0 | 0 | 0 | 0.07 | 0.938 | 0.619 | 0.758 | 0.993 |
| DNAJA2 | HSPA8 | 0 | 0.431 | 0 | 0.138 | 0.656 | 0.441 | 0.507 | 0.945 |
| DNAJA2 | HSPA2 | 0 | 0.432 | 0 | 0.138 | 0.656 | 0.538 | 0.291 | 0.934 |
| DNAJA2 | HSP90AA1 | 0 | 0 | 0 | 0.16 | 0.632 | 0.503 | 0.312 | 0.88 |
| DNAJA2 | DNAJB1 | 0 | 0.385 | 0.807 | 0.158 | 0.625 | 0.503 | 0.494 | 0.855 |
| DNAJA2 | HSPH1 | 0 | 0.399 | 0 | 0.322 | 0.698 | 0.176 | 0.404 | 0.928 |
| DNAJA2 | HSPA5 | 0 | 0.436 | 0 | 0.135 | 0.214 | 0.176 | 0.33 | 0.749 |
| DNAJB1 | HSPA8 | 0 | 0.417 | 0 | 0.444 | 0.487 | 0.401 | 0.67 | 0.961 |
| DNAJB1 | HSPA2 | 0 | 0.42 | 0 | 0.444 | 0.487 | 0.481 | 0.287 | 0.927 |
| DNAJB1 | CANX | 0 | 0 | 0 | 0.069 | 0.147 | 0 | 0.331 | 0.422 |
| DNAJB1 | HSP90AA1 | 0 | 0 | 0 | 0.611 | 0.354 | 0.437 | 0.668 | 0.946 |
| DNAJB1 | VCP | 0 | 0 | 0 | 0.339 | 0.051 | 0 | 0.272 | 0.503 |
| DNAJB1 | PGM1 | 0 | 0 | 0 | 0.546 | 0 | 0 | 0 | 0.546 |
| DNAJB1 | CRYAB | 0 | 0 | 0 | 0.073 | 0.071 | 0.516 | 0.414 | 0.723 |
| DNAJB1 | DNAJA2 | 0 | 0.385 | 0.807 | 0.158 | 0.625 | 0.503 | 0.494 | 0.855 |
| DNAJB1 | HSPA5 | 0 | 0.413 | 0 | 0.364 | 0.174 | 0.573 | 0.436 | 0.912 |
| DNAJB1 | HSPH1 | 0 | 0.313 | 0 | 0.192 | 0.514 | 0.383 | 0.645 | 0.93 |
| EDEM3 | HSPA8 | 0 | 0 | 0 | 0.059 | 0.42 | 0 | 0 | 0.43 |
| EDEM3 | HSPH1 | 0 | 0 | 0 | 0 | 0.484 | 0 | 0.064 | 0.496 |
| EDEM3 | UGGT1 | 0 | 0 | 0 | 0.409 | 0 | 0 | 0.444 | 0.657 |
| EDEM3 | HSPA5 | 0 | 0 | 0 | 0.059 | 0.484 | 0 | 0.376 | 0.671 |
| EDEM3 | CANX | 0 | 0 | 0 | 0.063 | 0.484 | 0 | 0.639 | 0.81 |
| EDEM3 | SEL1L | 0 | 0 | 0 | 0.116 | 0.488 | 0 | 0.732 | 0.868 |
| ENO1 | HSPA8 | 0 | 0 | 0 | 0.109 | 0.125 | 0 | 0.315 | 0.419 |
| ENO1 | LDHAL6B | 0.081 | 0 | 0 | 0.202 | 0.368 | 0.178 | 0.222 | 0.649 |
| ENO1 | ALDOA | 0 | 0 | 0 | 0.407 | 0.22 | 0.659 | 0.554 | 0.92 |
| ENO1 | PGAM1 | 0.1 | 0 | 0 | 0.557 | 0.209 | 0.793 | 0.29 | 0.945 |
| ENO1 | ENO3 | 0 | 0.449 | 0.981 | 0 | 0.571 | 0.423 | 0.341 | 0.745 |
| ENO1 | GAPDH | 0.101 | 0 | 0 | 0.587 | 0.438 | 0 | 0.466 | 0.873 |
| ENO1 | SAR1B | 0 | 0 | 0 | 0.338 | 0 | 0 | 0.156 | 0.417 |
| ENO1 | PGM1 | 0 | 0 | 0 | 0.633 | 0.219 | 0 | 0.287 | 0.777 |
| ENO1 | LDHA | 0.081 | 0 | 0 | 0.202 | 0.428 | 0.178 | 0.602 | 0.837 |
| ENO1 | PGAM2 | 0.1 | 0 | 0 | 0.557 | 0.209 | 0.793 | 0.29 | 0.945 |
| ENO1 | PK | 0.113 | 0 | 0 | 0.636 | 0.094 | 0.952 | 0.486 | 0.991 |
| ENO3 | LDHAL6B | 0.081 | 0 | 0 | 0.202 | 0.368 | 0.178 | 0.233 | 0.654 |
| ENO3 | ALDOA | 0 | 0 | 0 | 0.407 | 0.22 | 0.659 | 0.454 | 0.902 |
| ENO3 | PGAM1 | 0.1 | 0 | 0 | 0.557 | 0.425 | 0.935 | 0.29 | 0.987 |
| ENO3 | CRYAB | 0 | 0 | 0 | 0.064 | 0.047 | 0 | 0.389 | 0.407 |
| ENO3 | SAR1B | 0 | 0 | 0 | 0.338 | 0 | 0 | 0.156 | 0.417 |
| ENO3 | ENO1 | 0 | 0.449 | 0.981 | 0 | 0.571 | 0.423 | 0.341 | 0.745 |
| ENO3 | LDHA | 0.081 | 0 | 0 | 0.202 | 0.428 | 0.178 | 0.44 | 0.771 |
| ENO3 | PGM1 | 0 | 0 | 0 | 0.633 | 0.219 | 0 | 0.342 | 0.794 |
| ENO3 | GAPDH | 0.101 | 0 | 0 | 0.587 | 0.438 | 0 | 0.283 | 0.83 |
| ENO3 | PGAM2 | 0.1 | 0 | 0 | 0.557 | 0.503 | 0.945 | 0.7 | 0.996 |
| ENO3 | PK | 0.113 | 0 | 0 | 0.636 | 0.155 | 0.984 | 0.511 | 0.997 |
| FCGR1A | ARPC5 | 0 | 0 | 0 | 0.059 | 0 | 0.6 | 0 | 0.607 |
| FCGR1A | PLA2G4A | 0 | 0 | 0 | 0.063 | 0 | 0.43 | 0 | 0.443 |
| FCGR1A | FCGR3A | 0 | 0 | 0.854 | 0.583 | 0.306 | 0.841 | 0.731 | 0.955 |
| FCGR1A | FCGR2B | 0 | 0 | 0.805 | 0.117 | 0 | 0.277 | 0.791 | 0.435 |
| FCGR1A | ARPC1B | 0 | 0 | 0 | 0.062 | 0 | 0.6 | 0 | 0.608 |
| FCGR2B | ARPC5 | 0 | 0 | 0 | 0.06 | 0 | 0.541 | 0 | 0.55 |
| FCGR2B | FCGR1A | 0 | 0 | 0.805 | 0.117 | 0 | 0.277 | 0.791 | 0.435 |
| FCGR2B | ARPC1B | 0 | 0 | 0 | 0.061 | 0 | 0.541 | 0 | 0.55 |
| FCGR3A | ARPC1B | 0 | 0 | 0 | 0.063 | 0 | 0.604 | 0 | 0.613 |
| FCGR3A | FCGR1A | 0 | 0 | 0.854 | 0.583 | 0.306 | 0.841 | 0.731 | 0.955 |
| GAPDH | HSPA8 | 0.042 | 0 | 0 | 0.109 | 0.366 | 0.343 | 0.509 | 0.794 |
| GAPDH | HSPA2 | 0.042 | 0 | 0 | 0.109 | 0.203 | 0.125 | 0.289 | 0.499 |
| GAPDH | CANX | 0 | 0 | 0 | 0.059 | 0 | 0 | 0.523 | 0.532 |
| GAPDH | MAPK3 | 0 | 0 | 0 | 0 | 0.115 | 0 | 0.601 | 0.632 |
| GAPDH | HSP90AA1 | 0 | 0 | 0 | 0.104 | 0.197 | 0 | 0.434 | 0.557 |
| GAPDH | ALDOA | 0.107 | 0 | 0 | 0.554 | 0.367 | 0.98 | 0.416 | 0.996 |
| GAPDH | PGAM1 | 0 | 0 | 0 | 0.574 | 0.154 | 0.192 | 0.285 | 0.763 |
| GAPDH | ENO3 | 0.101 | 0 | 0 | 0.587 | 0.438 | 0 | 0.283 | 0.83 |
| GAPDH | CFL1 | 0 | 0 | 0 | 0.14 | 0.09 | 0 | 0.325 | 0.425 |
| GAPDH | PGM1 | 0.095 | 0 | 0 | 0.259 | 0.153 | 0 | 0.205 | 0.488 |
| GAPDH | LDHA | 0.083 | 0 | 0 | 0.161 | 0.12 | 0 | 0.479 | 0.6 |
| GAPDH | HSPA5 | 0.042 | 0 | 0 | 0.069 | 0.15 | 0.082 | 0.647 | 0.71 |
| GAPDH | PGAM2 | 0 | 0 | 0 | 0.574 | 0.154 | 0.192 | 0.384 | 0.796 |
| GAPDH | PK | 0.113 | 0 | 0 | 0.622 | 0.185 | 0 | 0.442 | 0.827 |
| GAPDH | ENO1 | 0.101 | 0 | 0 | 0.587 | 0.438 | 0 | 0.466 | 0.873 |
| GSN | CFL1 | 0 | 0 | 0 | 0.06 | 0.051 | 0 | 0.612 | 0.623 |
| HERPUD1 | SEL1L | 0 | 0 | 0 | 0.065 | 0.103 | 0 | 0.458 | 0.505 |
| HERPUD1 | DERL1 | 0 | 0 | 0 | 0 | 0.123 | 0 | 0.485 | 0.529 |
| HERPUD1 | VCP | 0 | 0 | 0 | 0.047 | 0.199 | 0 | 0.279 | 0.401 |
| HERPUD1 | PPP1R15A | 0 | 0 | 0 | 0 | 0 | 0 | 0.445 | 0.445 |
| HERPUD1 | HSPA5 | 0 | 0 | 0 | 0.206 | 0 | 0 | 0.567 | 0.641 |
| HK3 | PK | 0 | 0 | 0 | 0.135 | 0.065 | 0 | 0.394 | 0.467 |
| HK3 | ALDOA | 0 | 0 | 0 | 0.066 | 0 | 0.6 | 0.287 | 0.71 |
| HK3 | PGM1 | 0 | 0 | 0 | 0.113 | 0 | 0.984 | 0.319 | 0.989 |
| HSP90AA1 | HSPA8 | 0 | 0 | 0 | 0.546 | 0.565 | 0.506 | 0.822 | 0.98 |
| HSP90AA1 | MAP3K5 | 0 | 0 | 0 | 0 | 0.794 | 0 | 0.144 | 0.816 |
| HSP90AA1 | HSPA2 | 0 | 0 | 0 | 0.546 | 0.565 | 0.48 | 0.313 | 0.92 |
| HSP90AA1 | CANX | 0 | 0 | 0 | 0.225 | 0.153 | 0 | 0.43 | 0.593 |
| HSP90AA1 | MAPK3 | 0 | 0 | 0 | 0.059 | 0.306 | 0.526 | 0.313 | 0.758 |
| HSP90AA1 | PK | 0 | 0 | 0 | 0.089 | 0.153 | 0 | 0.329 | 0.437 |
| HSP90AA1 | WASF3 | 0 | 0 | 0 | 0 | 0 | 0.311 | 0.228 | 0.445 |
| HSP90AA1 | GAPDH | 0 | 0 | 0 | 0.104 | 0.197 | 0 | 0.434 | 0.557 |
| HSP90AA1 | CRYAB | 0 | 0 | 0 | 0.067 | 0.155 | 0.523 | 0.457 | 0.768 |
| HSP90AA1 | VCP | 0 | 0 | 0 | 0.158 | 0.185 | 0.437 | 0.493 | 0.777 |
| HSP90AA1 | DNAJA2 | 0 | 0 | 0 | 0.16 | 0.632 | 0.503 | 0.312 | 0.88 |
| HSP90AA1 | HSPH1 | 0 | 0 | 0 | 0.595 | 0.511 | 0.264 | 0.358 | 0.893 |
| HSP90AA1 | DNAJB1 | 0 | 0 | 0 | 0.611 | 0.354 | 0.437 | 0.668 | 0.946 |
| HSP90AA1 | HSPA5 | 0 | 0 | 0 | 0.67 | 0.352 | 0.264 | 0.738 | 0.953 |
| HSPA2 | HSPA8 | 0 | 0.449 | 0.983 | 0 | 0.638 | 0.399 | 0.442 | 0.776 |
| HSPA2 | CANX | 0 | 0 | 0 | 0.115 | 0.198 | 0.074 | 0.246 | 0.438 |
| HSPA2 | GAPDH | 0.042 | 0 | 0 | 0.109 | 0.203 | 0.125 | 0.289 | 0.499 |
| HSPA2 | MAPK3 | 0 | 0 | 0 | 0 | 0.11 | 0.444 | 0.089 | 0.509 |
| HSPA2 | CRYAB | 0 | 0 | 0 | 0.069 | 0.15 | 0.431 | 0.205 | 0.594 |
| HSPA2 | MAPK9 | 0 | 0 | 0 | 0 | 0 | 0.6 | 0.072 | 0.612 |
| HSPA2 | VCP | 0.043 | 0 | 0 | 0.102 | 0.563 | 0 | 0.309 | 0.705 |
| HSPA2 | HSPA5 | 0 | 0.448 | 0.965 | 0.259 | 0.493 | 0.502 | 0.394 | 0.802 |
| HSPA2 | HSPH1 | 0 | 0.433 | 0.728 | 0.287 | 0.628 | 0.64 | 0.339 | 0.915 |
| HSPA2 | HSP90AA1 | 0 | 0 | 0 | 0.546 | 0.565 | 0.48 | 0.313 | 0.92 |
| HSPA2 | DNAJB1 | 0 | 0.42 | 0 | 0.444 | 0.487 | 0.481 | 0.287 | 0.927 |
| HSPA2 | DNAJA2 | 0 | 0.432 | 0 | 0.138 | 0.656 | 0.538 | 0.291 | 0.934 |
| HSPA5 | HSPA8 | 0 | 0.448 | 0.965 | 0.259 | 0.493 | 0.421 | 0.715 | 0.772 |
| HSPA5 | MAP3K5 | 0 | 0 | 0 | 0.06 | 0.043 | 0 | 0.444 | 0.456 |
| HSPA5 | HSPA2 | 0 | 0.448 | 0.965 | 0.259 | 0.493 | 0.502 | 0.394 | 0.802 |
| HSPA5 | EDEM3 | 0 | 0 | 0 | 0.059 | 0.484 | 0 | 0.376 | 0.671 |
| HSPA5 | SEL1L | 0.044 | 0 | 0 | 0.066 | 0.429 | 0 | 0.687 | 0.819 |
| HSPA5 | CANX | 0 | 0 | 0 | 0.724 | 0.688 | 0.311 | 0.835 | 0.988 |
| HSPA5 | MAPK3 | 0 | 0 | 0 | 0.058 | 0.11 | 0.187 | 0.403 | 0.538 |
| HSPA5 | HSP90AA1 | 0 | 0 | 0 | 0.67 | 0.352 | 0.264 | 0.738 | 0.953 |
| HSPA5 | DNAJB1 | 0 | 0.413 | 0 | 0.364 | 0.174 | 0.573 | 0.436 | 0.912 |
| HSPA5 | GAPDH | 0.042 | 0 | 0 | 0.069 | 0.15 | 0.082 | 0.647 | 0.71 |
| HSPA5 | DERL1 | 0 | 0 | 0 | 0.099 | 0.077 | 0 | 0.489 | 0.538 |
| HSPA5 | HSPH1 | 0 | 0.434 | 0.73 | 0.408 | 0.103 | 0.638 | 0.444 | 0.835 |
| HSPA5 | VCP | 0.043 | 0 | 0 | 0.128 | 0.15 | 0 | 0.702 | 0.76 |
| HSPA5 | PPP1R15A | 0 | 0 | 0 | 0 | 0.051 | 0 | 0.697 | 0.7 |
| HSPA5 | CRYAB | 0 | 0 | 0 | 0.053 | 0.093 | 0.137 | 0.332 | 0.438 |
| HSPA5 | SAR1B | 0 | 0 | 0 | 0.187 | 0 | 0 | 0.359 | 0.456 |
| HSPA5 | UGGT1 | 0 | 0 | 0 | 0.155 | 0.11 | 0 | 0.358 | 0.475 |
| HSPA5 | MARCH6 | 0 | 0 | 0 | 0 | 0.051 | 0 | 0.546 | 0.55 |
| HSPA5 | SEC24A | 0 | 0 | 0 | 0.464 | 0.095 | 0 | 0.31 | 0.636 |
| HSPA5 | HERPUD1 | 0 | 0 | 0 | 0.206 | 0 | 0 | 0.567 | 0.641 |
| HSPA5 | DNAJA2 | 0 | 0.436 | 0 | 0.135 | 0.214 | 0.176 | 0.33 | 0.749 |
| HSPA8 | ENO1 | 0 | 0 | 0 | 0.109 | 0.125 | 0 | 0.315 | 0.419 |
| HSPA8 | EDEM3 | 0 | 0 | 0 | 0.059 | 0.42 | 0 | 0 | 0.43 |
| HSPA8 | CANX | 0 | 0 | 0 | 0.115 | 0.198 | 0.074 | 0.35 | 0.515 |
| HSPA8 | LDHA | 0 | 0 | 0 | 0.068 | 0.092 | 0 | 0.487 | 0.527 |
| HSPA8 | MAPK3 | 0 | 0 | 0 | 0 | 0.11 | 0.471 | 0.089 | 0.533 |
| HSPA8 | MAPK9 | 0 | 0 | 0 | 0 | 0 | 0.6 | 0.072 | 0.612 |
| HSPA8 | PK | 0.056 | 0 | 0 | 0.065 | 0.065 | 0.054 | 0.654 | 0.68 |
| HSPA8 | CRYAB | 0 | 0 | 0 | 0.069 | 0.15 | 0.513 | 0.339 | 0.711 |
| HSPA8 | VCP | 0.043 | 0 | 0 | 0.102 | 0.563 | 0 | 0.44 | 0.761 |
| HSPA8 | HSPA5 | 0 | 0.448 | 0.965 | 0.259 | 0.493 | 0.421 | 0.715 | 0.772 |
| HSPA8 | HSPA2 | 0 | 0.449 | 0.983 | 0 | 0.638 | 0.399 | 0.442 | 0.776 |
| HSPA8 | GAPDH | 0.042 | 0 | 0 | 0.109 | 0.366 | 0.343 | 0.509 | 0.794 |
| HSPA8 | DNAJA2 | 0 | 0.431 | 0 | 0.138 | 0.656 | 0.441 | 0.507 | 0.945 |
| HSPA8 | DNAJB1 | 0 | 0.417 | 0 | 0.444 | 0.487 | 0.401 | 0.67 | 0.961 |
| HSPA8 | HSPH1 | 0 | 0.433 | 0.732 | 0.649 | 0.696 | 0.575 | 0.653 | 0.963 |
| HSPA8 | HSP90AA1 | 0 | 0 | 0 | 0.546 | 0.565 | 0.506 | 0.822 | 0.98 |
| HSPH1 | HSPA8 | 0 | 0.433 | 0.732 | 0.649 | 0.696 | 0.575 | 0.653 | 0.963 |
| HSPH1 | HSPA2 | 0 | 0.433 | 0.728 | 0.287 | 0.628 | 0.64 | 0.339 | 0.915 |
| HSPH1 | EDEM3 | 0 | 0 | 0 | 0 | 0.484 | 0 | 0.064 | 0.496 |
| HSPH1 | CANX | 0 | 0 | 0 | 0.116 | 0.198 | 0.074 | 0.256 | 0.446 |
| HSPH1 | HSP90AA1 | 0 | 0 | 0 | 0.595 | 0.511 | 0.264 | 0.358 | 0.893 |
| HSPH1 | DNAJB1 | 0 | 0.313 | 0 | 0.192 | 0.514 | 0.383 | 0.645 | 0.93 |
| HSPH1 | VCP | 0.043 | 0 | 0 | 0.137 | 0.154 | 0 | 0.26 | 0.413 |
| HSPH1 | HSPA5 | 0 | 0.434 | 0.73 | 0.408 | 0.103 | 0.638 | 0.444 | 0.835 |
| HSPH1 | DNAJA2 | 0 | 0.399 | 0 | 0.322 | 0.698 | 0.176 | 0.404 | 0.928 |
| LDHA | HSPA8 | 0 | 0 | 0 | 0.068 | 0.092 | 0 | 0.487 | 0.527 |
| LDHA | ALDOA | 0 | 0 | 0 | 0.114 | 0.136 | 0.6 | 0.66 | 0.882 |
| LDHA | ENO3 | 0.081 | 0 | 0 | 0.202 | 0.428 | 0.178 | 0.44 | 0.771 |
| LDHA | CFL2 | 0 | 0 | 0 | 0.067 | 0.393 | 0 | 0.073 | 0.429 |
| LDHA | GAPDH | 0.083 | 0 | 0 | 0.161 | 0.12 | 0 | 0.479 | 0.6 |
| LDHA | ENO1 | 0.081 | 0 | 0 | 0.202 | 0.428 | 0.178 | 0.602 | 0.837 |
| LDHA | CFL1 | 0 | 0 | 0 | 0.067 | 0.393 | 0 | 0.122 | 0.459 |
| LDHA | PGAM2 | 0 | 0 | 0 | 0.104 | 0.155 | 0 | 0.385 | 0.494 |
| LDHA | PGM1 | 0 | 0 | 0 | 0.139 | 0.258 | 0 | 0.569 | 0.7 |
| LDHA | PK | 0.103 | 0 | 0 | 0.125 | 0.204 | 0.938 | 0.815 | 0.991 |
| LDHAL6B | CFL2 | 0 | 0 | 0 | 0.067 | 0.393 | 0 | 0.047 | 0.413 |
| LDHAL6B | CFL1 | 0 | 0 | 0 | 0.067 | 0.393 | 0 | 0.047 | 0.413 |
| LDHAL6B | PGM1 | 0 | 0 | 0 | 0.139 | 0.215 | 0 | 0.295 | 0.481 |
| LDHAL6B | ENO1 | 0.081 | 0 | 0 | 0.202 | 0.368 | 0.178 | 0.222 | 0.649 |
| LDHAL6B | ENO3 | 0.081 | 0 | 0 | 0.202 | 0.368 | 0.178 | 0.233 | 0.654 |
| LDHAL6B | ALDOA | 0 | 0 | 0 | 0.111 | 0.136 | 0.6 | 0.333 | 0.767 |
| LDHAL6B | PK | 0.103 | 0 | 0 | 0.125 | 0.155 | 0.938 | 0.51 | 0.976 |
| MAN1A2 | UGGT1 | 0 | 0 | 0 | 0.229 | 0.049 | 0 | 0.258 | 0.408 |
| MAP3K5 | HSPA5 | 0 | 0 | 0 | 0.06 | 0.043 | 0 | 0.444 | 0.456 |
| MAP3K5 | CDC42 | 0 | 0 | 0 | 0 | 0 | 0.8 | 0 | 0.8 |
| MAP3K5 | HSP90AA1 | 0 | 0 | 0 | 0 | 0.794 | 0 | 0.144 | 0.816 |
| MAP3K5 | MAPK9 | 0 | 0 | 0.607 | 0 | 0 | 0.8 | 0.506 | 0.838 |
| MAPK3 | HSPA8 | 0 | 0 | 0 | 0 | 0.11 | 0.471 | 0.089 | 0.533 |
| MAPK3 | HSPA2 | 0 | 0 | 0 | 0 | 0.11 | 0.444 | 0.089 | 0.509 |
| MAPK3 | MAPK9 | 0 | 0.404 | 0.88 | 0.077 | 0.092 | 0.411 | 0.526 | 0.518 |
| MAPK3 | CRYAB | 0 | 0 | 0 | 0 | 0.149 | 0.272 | 0.11 | 0.4 |
| MAPK3 | PLD1 | 0 | 0 | 0 | 0.048 | 0.078 | 0.413 | 0.089 | 0.467 |
| MAPK3 | CDC42 | 0 | 0 | 0 | 0 | 0.203 | 0.202 | 0.246 | 0.479 |
| MAPK3 | PK | 0 | 0 | 0 | 0.062 | 0.117 | 0 | 0.44 | 0.495 |
| MAPK3 | HSPA5 | 0 | 0 | 0 | 0.058 | 0.11 | 0.187 | 0.403 | 0.538 |
| MAPK3 | GAPDH | 0 | 0 | 0 | 0 | 0.115 | 0 | 0.601 | 0.632 |
| MAPK3 | HSP90AA1 | 0 | 0 | 0 | 0.059 | 0.306 | 0.526 | 0.313 | 0.758 |
| MAPK3 | SPHK1 | 0 | 0 | 0 | 0.046 | 0.042 | 0.804 | 0.234 | 0.845 |
| MAPK3 | PLA2G4A | 0 | 0 | 0 | 0 | 0.159 | 0.815 | 0.146 | 0.855 |
| MAPK3 | NCF1 | 0 | 0 | 0 | 0 | 0.121 | 0.822 | 0.243 | 0.871 |
| MAPK9 | HSPA8 | 0 | 0 | 0 | 0 | 0 | 0.6 | 0.072 | 0.612 |
| MAPK9 | MAP3K5 | 0 | 0 | 0.607 | 0 | 0 | 0.8 | 0.506 | 0.838 |
| MAPK9 | HSPA2 | 0 | 0 | 0 | 0 | 0 | 0.6 | 0.072 | 0.612 |
| MAPK9 | PK | 0 | 0 | 0 | 0.047 | 0 | 0 | 0.437 | 0.44 |
| MAPK9 | MAPK3 | 0 | 0.404 | 0.88 | 0.077 | 0.092 | 0.411 | 0.526 | 0.518 |
| MAPK9 | CDC42 | 0 | 0 | 0 | 0.059 | 0.205 | 0.849 | 0.057 | 0.879 |
| MARCH6 | SEL1L | 0 | 0 | 0 | 0.062 | 0 | 0.277 | 0.529 | 0.652 |
| MARCH6 | DERL1 | 0 | 0 | 0 | 0 | 0.072 | 0.113 | 0.508 | 0.559 |
| MARCH6 | VCP | 0 | 0 | 0 | 0.061 | 0.913 | 0 | 0.69 | 0.972 |
| MARCH6 | HSPA5 | 0 | 0 | 0 | 0 | 0.051 | 0 | 0.546 | 0.55 |
| NCF1 | MAPK3 | 0 | 0 | 0 | 0 | 0.121 | 0.822 | 0.243 | 0.871 |
| NCF1 | PLA2G4A | 0 | 0 | 0 | 0 | 0.436 | 0 | 0.444 | 0.673 |
| NCF1 | CDC42 | 0 | 0 | 0 | 0.059 | 0.316 | 0.199 | 0.147 | 0.501 |
| PGAM1 | ALDOA | 0.104 | 0 | 0 | 0.158 | 0.12 | 0.8 | 0.236 | 0.88 |
| PGAM1 | GAPDH | 0 | 0 | 0 | 0.574 | 0.154 | 0.192 | 0.285 | 0.763 |
| PGAM1 | PK | 0.106 | 0 | 0 | 0.646 | 0 | 0.139 | 0.359 | 0.801 |
| PGAM1 | PGAM2 | 0 | 0.449 | 0.98 | 0 | 0.203 | 0.883 | 0.516 | 0.904 |
| PGAM1 | ENO1 | 0.1 | 0 | 0 | 0.557 | 0.209 | 0.793 | 0.29 | 0.945 |
| PGAM1 | ENO3 | 0.1 | 0 | 0 | 0.557 | 0.425 | 0.935 | 0.29 | 0.987 |
| PGAM2 | ALDOA | 0.104 | 0 | 0 | 0.158 | 0.176 | 0.8 | 0.435 | 0.916 |
| PGAM2 | PGAM1 | 0 | 0.449 | 0.98 | 0 | 0.203 | 0.883 | 0.516 | 0.904 |
| PGAM2 | ENO3 | 0.1 | 0 | 0 | 0.557 | 0.503 | 0.945 | 0.7 | 0.996 |
| PGAM2 | GAPDH | 0 | 0 | 0 | 0.574 | 0.154 | 0.192 | 0.384 | 0.796 |
| PGAM2 | ENO1 | 0.1 | 0 | 0 | 0.557 | 0.209 | 0.793 | 0.29 | 0.945 |
| PGAM2 | LDHA | 0 | 0 | 0 | 0.104 | 0.155 | 0 | 0.385 | 0.494 |
| PGAM2 | PK | 0.106 | 0 | 0 | 0.646 | 0 | 0.116 | 0.501 | 0.841 |
| PGM1 | LDHAL6B | 0 | 0 | 0 | 0.139 | 0.215 | 0 | 0.295 | 0.481 |
| PGM1 | HK3 | 0 | 0 | 0 | 0.113 | 0 | 0.984 | 0.319 | 0.989 |
| PGM1 | ALDOA | 0 | 0 | 0 | 0.298 | 0.045 | 0.851 | 0.287 | 0.919 |
| PGM1 | DNAJB1 | 0 | 0 | 0 | 0.546 | 0 | 0 | 0 | 0.546 |
| PGM1 | ENO3 | 0 | 0 | 0 | 0.633 | 0.219 | 0 | 0.342 | 0.794 |
| PGM1 | GAPDH | 0.095 | 0 | 0 | 0.259 | 0.153 | 0 | 0.205 | 0.488 |
| PGM1 | ENO1 | 0 | 0 | 0 | 0.633 | 0.219 | 0 | 0.287 | 0.777 |
| PGM1 | LDHA | 0 | 0 | 0 | 0.139 | 0.258 | 0 | 0.569 | 0.7 |
| PGM1 | PK | 0.107 | 0 | 0 | 0.162 | 0 | 0 | 0.313 | 0.44 |
| PK | HSPA8 | 0.056 | 0 | 0 | 0.065 | 0.065 | 0.054 | 0.654 | 0.68 |
| PK | MAPK9 | 0 | 0 | 0 | 0.047 | 0 | 0 | 0.437 | 0.44 |
| PK | LDHAL6B | 0.103 | 0 | 0 | 0.125 | 0.155 | 0.938 | 0.51 | 0.976 |
| PK | HK3 | 0 | 0 | 0 | 0.135 | 0.065 | 0 | 0.394 | 0.467 |
| PK | MAPK3 | 0 | 0 | 0 | 0.062 | 0.117 | 0 | 0.44 | 0.495 |
| PK | HSP90AA1 | 0 | 0 | 0 | 0.089 | 0.153 | 0 | 0.329 | 0.437 |
| PK | ALDOA | 0.121 | 0 | 0 | 0.275 | 0.106 | 0.8 | 0.608 | 0.947 |
| PK | PGAM1 | 0.106 | 0 | 0 | 0.646 | 0 | 0.139 | 0.359 | 0.801 |
| PK | ENO3 | 0.113 | 0 | 0 | 0.636 | 0.155 | 0.984 | 0.511 | 0.997 |
| PK | GAPDH | 0.113 | 0 | 0 | 0.622 | 0.185 | 0 | 0.442 | 0.827 |
| PK | ENO1 | 0.113 | 0 | 0 | 0.636 | 0.094 | 0.952 | 0.486 | 0.991 |
| PK | LDHA | 0.103 | 0 | 0 | 0.125 | 0.204 | 0.938 | 0.815 | 0.991 |
| PK | PGM1 | 0.107 | 0 | 0 | 0.162 | 0 | 0 | 0.313 | 0.44 |
| PK | PGAM2 | 0.106 | 0 | 0 | 0.646 | 0 | 0.116 | 0.501 | 0.841 |
| PLA2G4A | MAPK3 | 0 | 0 | 0 | 0 | 0.159 | 0.815 | 0.146 | 0.855 |
| PLA2G4A | FCGR1A | 0 | 0 | 0 | 0.063 | 0 | 0.43 | 0 | 0.443 |
| PLA2G4A | NCF1 | 0 | 0 | 0 | 0 | 0.436 | 0 | 0.444 | 0.673 |
| PLA2G4A | PLD1 | 0 | 0 | 0 | 0.048 | 0 | 0.65 | 0.401 | 0.782 |
| PLD1 | MAPK3 | 0 | 0 | 0 | 0.048 | 0.078 | 0.413 | 0.089 | 0.467 |
| PLD1 | SPHK1 | 0.041 | 0 | 0 | 0.061 | 0 | 0.8 | 0.196 | 0.835 |
| PLD1 | PLA2G4A | 0 | 0 | 0 | 0.048 | 0 | 0.65 | 0.401 | 0.782 |
| PLD1 | CDC42 | 0 | 0 | 0 | 0 | 0.148 | 0.31 | 0.149 | 0.456 |
| PLD1 | ALDH3B1 | 0 | 0 | 0 | 0 | 0 | 0.462 | 0.042 | 0.462 |
| PPP1R15A | HERPUD1 | 0 | 0 | 0 | 0 | 0 | 0 | 0.445 | 0.445 |
| PPP1R15A | HSPA5 | 0 | 0 | 0 | 0 | 0.051 | 0 | 0.697 | 0.7 |
| SAR1B | CANX | 0 | 0 | 0 | 0.067 | 0 | 0.411 | 0.205 | 0.524 |
| SAR1B | ENO3 | 0 | 0 | 0 | 0.338 | 0 | 0 | 0.156 | 0.417 |
| SAR1B | ENO1 | 0 | 0 | 0 | 0.338 | 0 | 0 | 0.156 | 0.417 |
| SAR1B | HSPA5 | 0 | 0 | 0 | 0.187 | 0 | 0 | 0.359 | 0.456 |
| SAR1B | SEC24A | 0 | 0 | 0 | 0.078 | 0.713 | 0.502 | 0.589 | 0.938 |
| SEC24A | CANX | 0 | 0 | 0 | 0.157 | 0.045 | 0.363 | 0.429 | 0.667 |
| SEC24A | HSPA5 | 0 | 0 | 0 | 0.464 | 0.095 | 0 | 0.31 | 0.636 |
| SEC24A | SAR1B | 0 | 0 | 0 | 0.078 | 0.713 | 0.502 | 0.589 | 0.938 |
| SEL1L | EDEM3 | 0 | 0 | 0 | 0.116 | 0.488 | 0 | 0.732 | 0.868 |
| SEL1L | UGGT1 | 0 | 0 | 0 | 0.116 | 0 | 0 | 0.407 | 0.453 |
| SEL1L | HERPUD1 | 0 | 0 | 0 | 0.065 | 0.103 | 0 | 0.458 | 0.505 |
| SEL1L | MARCH6 | 0 | 0 | 0 | 0.062 | 0 | 0.277 | 0.529 | 0.652 |
| SEL1L | CANX | 0 | 0 | 0 | 0.072 | 0.041 | 0 | 0.643 | 0.654 |
| SEL1L | HSPA5 | 0.044 | 0 | 0 | 0.066 | 0.429 | 0 | 0.687 | 0.819 |
| SEL1L | DERL1 | 0 | 0 | 0 | 0.087 | 0.495 | 0.603 | 0.832 | 0.965 |
| SEL1L | VCP | 0.044 | 0 | 0 | 0.097 | 0.793 | 0.61 | 0.652 | 0.971 |
| SPHK1 | MAPK3 | 0 | 0 | 0 | 0.046 | 0.042 | 0.804 | 0.234 | 0.845 |
| SPHK1 | PLD1 | 0.041 | 0 | 0 | 0.061 | 0 | 0.8 | 0.196 | 0.835 |
| UBXN6 | DERL1 | 0 | 0 | 0 | 0.059 | 0.103 | 0 | 0.352 | 0.405 |
| UBXN6 | UGGT1 | 0 | 0 | 0 | 0.064 | 0.103 | 0 | 0.444 | 0.492 |
| UBXN6 | VCP | 0 | 0 | 0 | 0.063 | 0.912 | 0.8 | 0.69 | 0.994 |
| UGGT1 | EDEM3 | 0 | 0 | 0 | 0.409 | 0 | 0 | 0.444 | 0.657 |
| UGGT1 | SEL1L | 0 | 0 | 0 | 0.116 | 0 | 0 | 0.407 | 0.453 |
| UGGT1 | CANX | 0 | 0 | 0 | 0.142 | 0.342 | 0 | 0.69 | 0.809 |
| UGGT1 | UBXN6 | 0 | 0 | 0 | 0.064 | 0.103 | 0 | 0.444 | 0.492 |
| UGGT1 | HSPA5 | 0 | 0 | 0 | 0.155 | 0.11 | 0 | 0.358 | 0.475 |
| UGGT1 | MAN1A2 | 0 | 0 | 0 | 0.229 | 0.049 | 0 | 0.258 | 0.408 |
| VCP | HSPA8 | 0.043 | 0 | 0 | 0.102 | 0.563 | 0 | 0.44 | 0.761 |
| VCP | HSPA2 | 0.043 | 0 | 0 | 0.102 | 0.563 | 0 | 0.309 | 0.705 |
| VCP | SEL1L | 0.044 | 0 | 0 | 0.097 | 0.793 | 0.61 | 0.652 | 0.971 |
| VCP | CANX | 0 | 0 | 0 | 0.091 | 0.776 | 0 | 0.58 | 0.907 |
| VCP | HSP90AA1 | 0 | 0 | 0 | 0.158 | 0.185 | 0.437 | 0.493 | 0.777 |
| VCP | DNAJB1 | 0 | 0 | 0 | 0.339 | 0.051 | 0 | 0.272 | 0.503 |
| VCP | UBXN6 | 0 | 0 | 0 | 0.063 | 0.912 | 0.8 | 0.69 | 0.994 |
| VCP | DERL1 | 0 | 0 | 0 | 0.07 | 0.938 | 0.619 | 0.758 | 0.993 |
| VCP | HSPH1 | 0.043 | 0 | 0 | 0.137 | 0.154 | 0 | 0.26 | 0.413 |
| VCP | HERPUD1 | 0 | 0 | 0 | 0.047 | 0.199 | 0 | 0.279 | 0.401 |
| VCP | HSPA5 | 0.043 | 0 | 0 | 0.128 | 0.15 | 0 | 0.702 | 0.76 |
| VCP | MARCH6 | 0 | 0 | 0 | 0.061 | 0.913 | 0 | 0.69 | 0.972 |
| WASF3 | ARPC5 | 0 | 0 | 0 | 0 | 0 | 0.538 | 0.234 | 0.63 |
| WASF3 | HSP90AA1 | 0 | 0 | 0 | 0 | 0 | 0.311 | 0.228 | 0.445 |
| WASF3 | CDC42 | 0 | 0 | 0 | 0 | 0.129 | 0.275 | 0.424 | 0.604 |
| WASF3 | ARPC1B | 0 | 0 | 0 | 0.059 | 0 | 0.538 | 0.167 | 0.606 |
