# Supplementary material for: Physiological and transcriptomic analysis dissects the molecular mechanism governing meat quality during postmortem aging in Hu sheep (Ovis aries)
Source: Front Nutr. 2024 Jan 5;10:1321938. doi: 10.3389/fnut.2023.1321938 (PMC10799347; doi:10.3389/fnut.2023.1321938)
Supplement: Supplementary file 2 [file Table_2.doc]

**Table S2.** Details of the clusters generated by the MCODE plugin.

| Cluster | Score | Node | Edge | Seed node | Node name |
| --- | --- | --- | --- | --- | --- |
| 1 | 7.4 | 11 | 37 | HSPA5 | HSPA1, HSPA5, HSPA2, HSPA8, HSP90AA1, DNAJB1, DNAJA2, VCP, PGM1, CANX, LDHA |
| 2 | 5.14 | 8 | 18 | GAPDH | GAPDH, PK, LDHAL6B, ALDOA, ENO1, CRYAB, MAPK3, ENO3 |
| 3 | 4 | 4 | 6 | FCGR1A | FCGR2B, FCGR1A, ARPC5, ARPC1B |
